# Deciphering the Molecular Basis of Cadmium Stress Adaptation in *Lentinula edodes*: Genome-Wide Identification of MAPK Cascade Genes and Their Functional Characterization

**DOI:** 10.3390/jof12100713

**Published:** 2026-09-23

**Authors:** Dandan Zhai, Xiaoxia Song, Tengye Luan, Xiaodong Shang, Meina He, Guirong Tang, Jianyu Liu, Yang Fu, Yansha Wu, Yu Li, Hailong Yu

**Affiliations:** 1 Engineering Research Centre of Chinese Ministry of Education for Edible and Medicinal Fungi, Jilin Agricultural University, Changchun 130118, China; zhaidandan0519@163.com (D.Z.);; 2 Institute of Edible Fungi, Shanghai Academy of Agricultural Sciences, National Engineering Research Center of Edible Fungi, Shanghai 201403, China; sxx8866@163.com (X.S.);

**Keywords:** *Lentinula edodes*, MAPK cascade components, gene family identification, cadmium stress, signal transduction pathway

## Abstract

Mitogen-activated protein kinase (MAPK) cascades play a central role in eukaryotic signal transduction, regulating diverse biological processes including development and stress responses. However, despite *Lentinula edodes* being the most widely cultivated edible mushroom worldwide, its MAPK cascade gene families remain uncharacterized, and their involvement in cadmium (Cd) stress responses is completely unknown. In this study, we performed genome-wide identification of MAPK cascade genes in *L. edodes*, uncovering 16 members comprising three *LeMAPKKK* genes, four *LeMAPKK* genes, and nine *LeMAPK* genes. Phylogenetic and homology analyses classified these genes into three putative MAPK cascade pathways. qRT-PCR analysis demonstrated strain-specific, concentration-dependent, and temporally dynamic expression patterns under Cd stress. Notably, four genes (*LeMAPK1*, *LeMAPK7*, *LeMAPKK2*, and *LeMAPKKK1*) exhibited consistent upregulation across both strains and high Cd concentrations (1 and 10 mg/L) at the 7 h time point. Based on these findings, we propose a working model for Cd stress response in *L. edodes* centered on these core regulatory genes. These findings may inform breeding strategies for low-Cd-accumulating cultivars and the optimization of cultivation practices to reduce Cd content, with potential implications for the sustainable development of the *L. edodes* industry and human health.

## 1. Introduction

The heavy metal cadmium (Cd), characterized by high toxicity, wide-ranging sources, and water solubility of its cationic form (Cd^2+^), poses a significant threat to environmental security, agricultural productivity, food safety, and human health [1]. *Lentinula edodes*, the most widely cultivated and consumed edible mushroom worldwide, is renowned for its exceptional nutritional value, distinctive flavor, and numerous health benefits [2]. However, like many cultivated mushrooms, *L. edodes* readily absorbs and accumulates heavy metals from the cultivation substrate, posing food safety concerns [3]. Beyond Cd accumulation, Cd stress also fundamentally affects the physiology of *L. edodes* [4]. Chen et al. reported that low Cd concentrations promote mycelial growth, while high-dose Cd exposure inhibits growth via cell wall damage and oxidative stress [5]. Wu et al. further revealed that different strains of *L. edodes* exhibit distinct response mechanisms when facing Cd stress, by employing either glutathione-mediated chelation or vacuolar sequestration, among other strategies [4]. While these studies have advanced our understanding of Cd responses in *L. edodes*, the upstream signaling pathways that regulate these processes remain largely unexplored.

The mitogen-activated protein kinase (MAPK) cascade is a central signaling module in eukaryotes, transducing extracellular stimuli into intracellular responses through sequential phosphorylation of three kinases: MAPK kinase kinase (MAPKKK), MAPK kinase (MAPKK) and a MAPK [6]. This three-tiered architecture enables signal amplification and integration of multiple inputs, making MAPK cascades ideal mediators of complex stress responses. Indeed, their involvement in Cd stress responses has been documented across diverse fungal taxa, including yeasts, filamentous fungi, and mushrooms. For instance, Cd exposure activates MAPK pathways in fission yeast [7]; in the filamentous fungus *Trichoderma atroviride*, MAPK signaling is essential for survival under high Cd concentrations [8]; and transcriptomic analysis of the mushroom *Morchella spongiola* identified MAPK signaling as a major Cd-responsive pathway [9]. Importantly, our own multi-omics data have provided direct evidence linking Cd stress to MAPK signaling in *L. edodes*. Transcriptomic and proteomic analyses comparing strains with divergent Cd stress responses revealed significant enrichment of MAPK pathway components among differentially expressed genes and proteins [4,10]. These findings collectively position MAPK cascades as prime candidates for mediating Cd responses in *L. edodes*.

To systematically characterize previously uninvestigated MAPK pathways in a given species, the essential first step is genome-wide identification of all MAPK, MAPKK, and MAPKKK family members through homology-based searches and phylogenetic analysis [11]. *Saccharomyces cerevisiae* serves as the primary reference, as its four functionally distinct MAPK pathways have been thoroughly characterized in both copy number and function [12,13]. By contrast, the class Agaricomycetes, to which *L. edodes* belongs, exhibits distinct features based on genome-wide predictions. Species in this class carry an average of 12.8 MAPK cascade components (MAPK+MAPKK+MAPKKK), higher than the fungal average of 11.2, with lineage-specific expansions predicted particularly in the Fus3/Kss1 and Slt2 subfamilies [11]. A representative example is *Schizophyllum commune* (also an Agaricomycete), which possesses six MAPKs, four MAPKKs, and four MAPKKKs, totaling 14 components and exceeding the class average [11]. These predictions suggest that Agaricomycetes may have evolved expanded and functionally diversified MAPK repertoires. However, systematic genome-wide identification and classification of MAPK, MAPKK, and MAPKKK gene families in *L. edodes*, and their alignment with yeast reference pathways, remains a critical knowledge gap.

To address this gap, we performed genome-wide identification of MAPK, MAPKK, and MAPKKK family members in *L. edodes* based on our previously assembled chromosome-level genome. Through homology searches and phylogenetic analyses with orthologs from *S. cerevisiae* and other fungi, we inferred the likely functional pathways of MAPK and MAPKK members. For MAPKKK members, which often exhibit greater sequence divergence, we extended the analysis to include orthologs from *Arabidopsis thaliana* to aid in accurate classification. We then examined their expression profiles under Cd stress using two strains (4606 and 4625), previously identified from a screen of 99 accessions and differing markedly in Cd accumulation (approximately three-fold) [14], across three concentrations (0.1, 1, and 10 mg/L) and two time points (0.5 h and 7 h). These conditions were selected because 0.1 mg/L captures the concentration at which differential accumulation between the two strains is most pronounced, while 1 and 10 mg/L represent moderate and severe stress. The 0.5 h time point corresponds to early signaling events, whereas 7 h reflects established transcriptional and physiological adjustments [4,10]. By integrating genome-wide identification, phylogenetic inference, and expression validation, this study provides a theoretical foundation for understanding MAPK-mediated Cd signal transduction in *L. edodes* and identifies actionable targets for modulating Cd stress responses. These findings may inform breeding strategies for low-Cd-accumulating cultivars and optimization of cultivation practices to reduce Cd content, with implications for the sustainable development of the *L. edodes* industry and human health.

## 2. Methods

### 2.1. Identification of MAPK, MAPKK, and MAPKKK Gene Families in L. edodes

The complete genome sequence and corresponding annotation files of the *L. edodes* L808 strain were retrieved from the NCBI database (Assembly accession: GCA_015476405.1). All protein sequences encoded by the genome were screened using TBtools-II (v2.458) software with the kinase domain PF00069 as the query, yielding candidate sequences [11,15]. These candidates were subjected to multiple sequence alignment using ClustalW in MEGA 11. Based on the alignments, sequences were manually retained only if they contained the complete characteristic motifs specific to each family: for MAPKKKs, the motifs -GTXX(W/Y)MAPE-, -GTPEFMAPE(L/V/M)(Y/F/L)-, or -G(T/S)PX(F/Y/W)MAPEV-; for MAPKKs, the motif S/TXXXXXS/T; and for MAPKs, the conserved TXY motif [16,17,18]. In addition, sequences encoding very short polypeptides were excluded, as such lengths fall well outside the typical size range of functional MAPK family members, as described in a previous genome-wide MAPK identification study in *Brassica rapa* [19].

### 2.2. Chromosomal Distribution

Based on the identification results described above, the chromosomal distribution of the MAPK, MAPKK, and MAPKKK gene families in *L. edodes* was further analyzed. According to their gene IDs and order of arrangement on chromosomes 1 to 10 in the L808 reference genome, the identified genes were systematically designated as *LeMAPK1* to *LeMAPKn*, *LeMAPKK1* to *LeMAPKKn*, and *LeMAPKKK1* to *LeMAPKKKn*, respectively (Table S1). Their locations within the L808 chromosomes were subsequently extracted and visualized using TBtools-II (v2.458).

### 2.3. Physicochemical Properties and Subcellular Localization Prediction

Physicochemical properties of all identified LeMAPK, LeMAPKK, and LeMAPKKK proteins were predicted using the Protein Parameter Calc tool in TBtools-II (v2.458), including amino acid number, molecular weight (kDa), theoretical isoelectric point (pI), grand average of hydropathicity (GRAVY), aliphatic index, and instability index. The resulting data were visualized using the ChiPlot online tool (https://www.chiplot.online/ (accessed on 11 December 2025)).

Subcellular localization was predicted using WoLF PSORT (https://wolfpsort.hgc.jp/ (accessed on 12 December 2025)) with the “fungi” organism option. Prediction scores for each localization category were visualized as heatmaps generated by ChiPlot.

### 2.4. Motif and Domain Analysis

Multiple sequence alignments of the LeMAPK, LeMAPKK, and LeMAPKKK protein sequences were performed using ClustalW in MEGA 11 with default parameters. Phylogenetic trees were constructed using the neighbor-joining (NJ) method with 1000 bootstrap replicates. Conserved motifs were predicted using MEME Suite (https://meme-suite.org/meme/tools/meme (accessed on 27 December 2025)) with motif number set to 10. To validate conserved domains, all protein sequences were submitted to the NCBI Batch CD-Search tool using default parameters, with “Concise” display, “Superfamily Only”, and “Include domain defline” selected. The phylogenetic tree, motif compositions, and domain architectures were integrated and visualized using ChiPlot.

### 2.5. Promoter Cis-Element Analysis

The 2000 bp upstream sequences from the transcription start sites of the *LeMAPK*, *LeMAPKK*, and *LeMAPKKK* genes were extracted using TBtools-II (v2.458). These putative promoter sequences were submitted to the PlantCARE online database (https://bioinformatics.psb.ugent.be/webtools/plantcare/html/ (accessed on 28 December 2025) for *cis*-acting element prediction. The raw prediction results were subsequently filtered to remove redundant information, and the filtered *cis*-element annotations were visualized as a distribution map of *cis*-acting elements using the ChiPlot online tool.

### 2.6. Phylogenetic and Evolutionary Analysis

To investigate the evolutionary relationships of the *L. edodes* MAPK cascade components, reference sequences were retrieved from the KEGG database for the yeast MAPK pathway (map04011), including MAPKKKs (Ste11, Bck1, Ssk2/22), MAPKKs (Ste7, Mkk1/2, Pbs2), and MAPKs (Fus3, Kss1, Hog1, Slt2). For the MAPK and MAPKK families, additional homologs were retrieved from species within the order Agaricales (Table S2 and S3). For the MAPKKK family, which exhibits greater sequence divergence, homologs were first retrieved from species within the phylum Basidiomycota (Table S4); sequences that could not be confidently classified were further analyzed with reference sequences from *A*. *thaliana*, representing the MEKK, Raf, and Zik subfamilies. All sequences were aligned using MUSCLE in MEGA 11 with default parameters, and phylogenetic trees were constructed using the neighbor-joining (NJ) method with 1000 bootstrap replicates.

### 2.7. Mycelial Cd Stress Treatment and qRT-PCR Expression Analysis

The *L. edodes* strains 4606 and 4625 were used in this study. Ten mycelial plugs (0.7 cm diameter) taken from the periphery of each pre-cultured plate were inoculated into 100 mL potato dextrose broth (PDB) to prepare liquid spawn. Cultures were incubated statically for 12 h, followed by shaking at 25 °C and 150 rpm in darkness for 7 d. The resulting liquid spawn was homogenized for 6 s, and 8 mL aliquots were transferred to fresh PDB. After another 12 h static incubation, cultures were shaken under the same conditions (25 °C, 150 rpm, dark) for 7 d. Based on previous studies [4,10,20], CdCl_2_ was added to final concentrations of 0 (control), 0.1, 1, and 10 mg/L. Mycelia were harvested at 0.5 h and 7 h post-treatment, filtered through non-woven fabric, rinsed with 100 mL distilled water, blotted dry, frozen in liquid nitrogen, and stored at −80 °C.

Total RNA was extracted from mycelial samples using a rapid plant total RNA isolation kit. cDNA was synthesized using a reverse transcription premix containing a gDNA remover. qRT-PCR was performed using the SYBR Green method, with the *L. edodes Actin* gene as an internal reference and untreated strains as controls [21]. Each sample included three biological replicates and three technical replicates. Relative expression levels were calculated using the 2^−ΔΔCt^ method. All pairwise comparisons were performed between each Cd-treated sample and its corresponding control (same strain, same time point). Raw *p*-values were obtained using the General Linear Model with simple contrast in SPSS 17.0. To control for false positives arising from multiple comparisons, all raw *p*-values were adjusted using the Benjamini–Hochberg (BH) false discovery rate (FDR) procedure in Microsoft Excel, with an adjusted *p*-value (q-value) < 0.05 considered statistically significant. Statistical analysis and graphical visualization were performed using GraphPad Prism 9.5.0 software.

## 3. Results

### 3.1. Identification and Basic Properties Analysis of LeMAPKs, LeMAPKKs, and LeMAPKKKs

A total of 17 MAPK cascade genes were identified in the *L. edodes* genome, comprising ten *LeMAPK* genes, four *LeMAPKK* genes, and three *LeMAPKKK* genes. One *LeMAPK* (KAF8827413.1) was excluded because its encoded protein contained only 100 amino acids, leaving nine LeMAPKs ranging from 363 to 733 amino acids in length. These genes exhibited marked variations in chromosomal distribution, while their encoded proteins differed in conserved motif composition, subcellular localization patterns, and physicochemical properties, as detailed below (Figure 1).

The nine *LeMAPK* genes were distributed across four chromosomes: *LeMAPK1* and *LeMAPK2* on chr1, *LeMAPK3* and *LeMAPK4* on chr2, *LeMAPK5* on chr3, and *LeMAPK6* through *LeMAPK9* forming a cluster on chr6 (Figure 1A). Their encoded proteins all contained the conserved TXY phosphorylation motif, present as TEY in eight members and TGY exclusively in LeMAPK2 (Figure 1B). Subcellular localization prediction revealed that all nine LeMAPKs were predominantly nuclear, with seven members exhibiting potential for nucleocytoplasmic shuttling (Figure 1C). The nine LeMAPKs varied in length (363–386 aa, except LeMAPK9 at 733 aa), molecular weight (40.9–43.9 kDa, 82.3 kDa for LeMAPK9), and pI (5.32–7.16) (Figure 1D–F). All were hydrophilic (negative GRAVY), most were unstable (instability index > 40 in seven members), and their aliphatic indices ranged from 76.51 to 93.29 (Figure 1G–I).

The four *LeMAPKK* genes were located on chr3 (*LeMAPKK1* and *LeMAPKK2*), chr4 (*LeMAPKK3*), and chr9 (*LeMAPKK4*) (Figure 1A). All four LeMAPKK proteins harbored the characteristic S-XXXXX-S conserved domain (Figure 1B). Subcellular localization patterns were diverse: LeMAPKK2 and LeMAPKK4 were predominantly mitochondrial, while LeMAPKK1 and LeMAPKK3 showed multi-compartmental or balanced distributions (Figure 1C). Compared to MAPKs, the MAPKK family exhibited broader ranges in length (396–582 aa), molecular weight (42.9–61.4 kDa), and pI (6.33–9.02) (Figure 1D–F). All members were hydrophilic, three were unstable, and aliphatic indices ranged from 83.16 to 93.76 (Figure 1G–I).

The three *LeMAPKKK* genes were located on chr2 (*LeMAPKKK1*) and chr6 (*LeMAPKKK2* and *LeMAPKKK3*) (Figure 1A). Their encoded proteins contained either the GTPYWMAPEV (LeMAPKKK1 and LeMAPKKK3) or GTPNWMAPEV (LeMAPKKK2) conserved domain (Figure 1B). Subcellular localization was markedly differentiated: LeMAPKKK1 was primarily mitochondrial, whereas LeMAPKKK2 and LeMAPKKK3 were predominantly nuclear (Figure 1C). The MAPKKK family exhibited the greatest variation among the three families, with protein lengths ranging from 726 to 1734 aa and molecular weights from 79.1 to 187.5 kDa (Figure 1D,E). All members had alkaline pI values (8.77–9.94), were hydrophilic, unstable (instability index > 40), and possessed relatively low aliphatic indices (64.12–70.18) (Figure 1F–I).

### 3.2. Phylogenetic, Motif, and Domain Analysis of LeMAPKs, LeMAPKKs, and LeMAPKKKs

Phylogenetic analysis clearly divided the *L. edodes* MAPK cascade genes into three independent clades with strong bootstrap support, corresponding to the LeMAPK, LeMAPKK, and LeMAPKKK families (Figure 2A). Conserved motif distribution and domain architectures were highly consistent with this clustering (Figure 2B,C).

The nine LeMAPKs constituted the largest clade with maximum bootstrap support (100%) (Figure 2A). All LeMAPKs contained Motif 2, Motif 3, and Motif 5, with Motif 2, Motif 6, and Motif 9 being specific to LeMAPKs (Figure 2B). Domain architectures varied among the nine LeMAPKs: LeMAPK9 contained STKc_MAPK; LeMAPK3 and LeMAPK4 contained STKc_ERK1_2_like; the remaining members contained the PKc_like superfamily as the core domain (Figure 2C).

The four LeMAPKKs also clustered into an independent clade with high bootstrap support (96%), and contained Motif 1, Motif 10, and Motif 7 (Figure 2A,B). Additionally, all four LeMAPKKs shared the PKc_like superfamily as the core domain, with LeMAPKK3 additionally containing PRK11901 superfamily (Figure 2C).

The three LeMAPKKKs formed a single clade with strong bootstrap support (85%), and contained Motif 4, Motif 1, Motif 10, Motif 7, and Motif 5 (Figure 2A,B). Domain architectures diverged among the three LeMAPKKKs: LeMAPKKK1 and LeMAPKKK3 shared PBD, the PHA03247 superfamily, and STKc_PAK, with LeMAPKKK1 additionally containing the PH_Cla4_Ste20 and PRK12323 superfamily; LeMAPKKK2 contained the PHA03247 superfamily, CH_SF, STKc_Cdc7_like, and C1 domains (Figure 2C).

### 3.3. Promoter Cis-Element Characteristics of LeMAPKs, LeMAPKKs, and LeMAPKKKs

*Cis*-acting element analysis of the 16 MAPK cascade genes revealed gene-specific distribution patterns in both element types and copy numbers (Figure 3). Light response elements were the most abundant in six *LeMAPK* genes (*LeMAPK2*, *LeMAPK4*, *LeMAPK5*, *LeMAPK7*, *LeMAPK8*, and *LeMAPK9*), two *LeMAPKK* genes (*LeMAPKK1* and *LeMAPKK2*), and *LeMAPKKK1*, with copy numbers exceeding ten in *LeMAPK2*, *LeMAPK7*, *LeMAPKK1*, and *LeMAPKK2*. MeJA response elements were the most abundant in three *LeMAPK* genes (*LeMAPK1*, *LeMAPK2*, and *LeMAPK3*), two *LeMAPKK* genes (*LeMAPKK3* and *LeMAPKK4*), and two *LeMAPKKK* genes (*LeMAPKKK2* and *LeMAPKKK3*), with six of these genes containing more than ten copies, except *LeMAPKKK3*. Low-temperature response elements were most abundant in *LeMAPK6* with five copies. In *LeMAPK8*, anaerobic induction elements were also among the most abundant, matching light response elements with three copies each. In *LeMAPK4*, gibberellin response elements were also among the most abundant, matching light response elements with three copies each.

### 3.4. Phylogenetic and Homology Analysis of LeMAPKs, LeMAPKKs, and LeMAPKKKs

The nine LeMAPKs were compared with 14 Fus3, 17 Kss1, nine Slt2, and eight Hog1 homologs from Agaricales species and *S. cerevisiae* (Figure 4A–D). Six LeMAPKs clustered with Fus3/Kss1 homologs but fell into distinct subclades: LeMAPK3 and LeMAPK4 each formed an independent branch, whereas LeMAPK1, LeMAPK6, LeMAPK7, and LeMAPK8 grouped together (Figure 4A,B). LeMAPK5 grouped with Slt2 (100% bootstrap support), and LeMAPK2 grouped with Hog1 (100% bootstrap support) (Figure 4C,D). In contrast, LeMAPK9 showed high sequence homology to Slt2 but clustered with it with only 54% bootstrap support, and did not associate with any other homolog group (Figure 4).

The four LeMAPKKs were aligned with six Mkk, 12 Ste7, and seven Pbs homologs from Agaricales and *S. cerevisiae* (Figure 4E–G). None of the LeMAPKKs clustered with Mkk homologs (Figure 4E). LeMAPKK2 clustered with Pbs2, whereas LeMAPKK1, LeMAPKK3, and LeMAPKK4 showed high sequence homology to Ste7 (Figure 4F,G).

Because the three LeMAPKKKs could not be resolved within Agaricales due to insufficient sequence divergence, the analysis was expanded to Basidiomycota, with *S. cerevisiae* and *Arabidopsis thaliana* as references (Figure 5A–C). The three LeMAPKKKs were compared with 25 Bck1, 47 Ste11, and 59 Ssk homologs from Basidiomycota and *S. cerevisiae*, along with four *A. thaliana* homologs for each group (Figure 5A–C). In the Bck1 comparison, all three LeMAPKKKs clustered with fungal and plant homologs (98% bootstrap support) (Figure 5A). In the Ste11 comparison, they clustered exclusively with the four *A. thaliana* homologs (Figure 5B). In the Ssk comparison, LeMAPKKK1 and LeMAPKKK3 associated with fungal homologs (79% bootstrap support), whereas LeMAPKKK2 showed no clear affinity to any group (Figure 5C). To further explore their potential classification, all three LeMAPKKKs were additionally compared with the full set of MAPKKK homologs from *A. thaliana*. They clustered together with a subset of *A. thaliana* MAPKKKs, albeit with moderate bootstrap support (78%) (Figure S1).

### 3.5. Cd-Induced Expression Patterns of LeMAPKs, LeMAPKKs, and LeMAPKKKs

qRT-PCR analysis revealed that the nine *LeMAPK* genes exhibited pronounced strain-specific and time-dependent transcriptional responses to Cd stress (Figure 6(A1–I1,A2–I2)). In strain 4606, no significant change in transcript abundance was detected at 0.5 h across any tested concentration. At 7 h, however, most genes showed upregulation, albeit with distinct concentration–response thresholds, whereas *LeMAPK5* was the sole member that was significantly repressed at the highest dose (10 mg/L). In contrast, strain 4625 mounted a more rapid and complex response: several genes were already activated at 0.5 h, and by 7 h, the expression patterns further resolved into two major categories: *LeMAPK1*, *LeMAPK6*, *LeMAPK7* and *LeMAPK8* were upregulated at all tested concentrations relative to their respective controls, while *LeMAPK2*, *LeMAPK3*, *LeMAPK4*, and *LeMAPK9* exhibited dose-dependent repression. Of particular note, *LeMAPK5* displayed a biphasic expression feature, with its direction of regulation reversing as the concentration increased (induced at the lowest dose but suppressed at higher doses). All other gene-treatment combinations showed transcript levels comparable to those of their respective controls.

The four *LeMAPKK* genes *also* exhibited strain-specific and time-dependent differential expression patterns in response to Cd stress (Figure 6(J1–M1,J2–M2)). Specifically, *LeMAPKK1* was significantly upregulated in strain 4606 under 10 mg/L Cd for 7 h and in strain 4625 under 1 mg/L Cd for 7 h; *LeMAPKK2* was upregulated in both strains after 7 h of exposure to 1 and 10 mg/L Cd; *LeMAPKK3* was downregulated only in strain 4625 under both 1 and 10 mg/L Cd for 7 h; and *LeMAPKK4* was upregulated in both strains after 0.5 h of 10 mg/L Cd treatment but downregulated in strain 4606 after 7 h of 10 mg/L Cd treatment and in strain 4625 after 7 h of 1 mg/L Cd treatment. All other treatments showed no significant difference compared to the control.

The three *LeMAPKKK* genes *also* exhibited strain-specific and time-dependent differential expression patterns in response to Cd stress (Figure 6(N1–P1,N2–P2)). The expression levels of the three *LeMAPKKK* genes remained unchanged in both strains after 0.5 h of Cd exposure across all tested concentrations. At 7 h under higher Cd concentrations (1 and 10 mg/L), the expression responses diverged among the three genes. *LeMAPKKK1* was significantly upregulated in both strains under 1 and 10 mg/L Cd treatments. *LeMAPKKK2* was significantly upregulated in strain 4606 under both 1 and 10 mg/L Cd, whereas in strain 4625, significant induction was observed only at 1 mg/L Cd. *LeMAPKKK3* showed no significant changes in strain 4606 at any concentration, but in strain 4625, it was significantly upregulated at 0.1 mg/L Cd while significantly downregulated at 1 and 10 mg/L Cd. All other treatments showed no significant difference compared to the control.

## 4. Discussion

### 4.1. Gene Family Identification and Classification of MAPK Cascade Components in L. edodes

A total of 16 MAPK cascade genes were identified in the *L. edodes* genome, exceeding the average of 12.8 components per species reported in Agaricomycetes [11]. It suggests that the MAPK cascade in *L. edodes* is generally conserved but has undergone lineage-specific expansion. Notably, among the nine LeMAPKs, six clustered with the yeast Fus3/Kss1 subfamily, whereas the average member count for this subfamily across the fungal kingdom is approximately 1.5 per species [11]. This fourfold expansion is particularly striking, suggesting that signaling pathways mediated by Fus3/Kss1 homologs (such as pheromone response and filamentous growth regulation) may have undergone functional divergence in *L. edodes*.

Among the nine LeMAPKs, LeMAPK9 stands out as exceptional. Its 733-amino-acid sequence is nearly twice the length of other LeMAPKs (363–386 aa, Figure 1D), and it is the only member containing both a unique STKc_MAPK domain and a salicylic acid-responsive element in its promoter (Figure 2C and Figure 3). This exceptional length and unique domain architecture may confer specialized capabilities in protein–protein interactions or substrate recognition, possibly involving stress- or development-specific signaling. However, its inability to cluster with any canonical subfamily (Figure 4A–D) makes its functional assignment uncertain. LeMAPK2 is the sole member carrying the TGY motif (Figure 1B), a hallmark of the Hog1 subfamily, and phylogenetic analysis confirmed its clustering with yeast Hog1 (Figure 4D). LeMAPK5 is the only member assigned to the Slt2 subfamily (Figure 4C). The distinct structural features of these two members, together with LeMAPK9, suggest that *L. edodes* possesses a functionally diversified MAPK repertoire, though experimental validation is needed to confirm their respective pathway assignments. Moreover, most LeMAPKs were predicted to be unstable (instability index > 40), which may contribute to the fine-tuning of signal intensity; analogously, in yeast, the stability of Msb2 protein differentially regulates the *fMAPK* and HOG pathways through the conformational integrity of its SEA domain [22].

The four LeMAPKKs showed a distinct distribution pattern. LeMAPKK2 clustered with the Pbs2 subfamily (Figure 4G), consistent with its role as the upstream kinase of Hog1-type MAPKs. LeMAPKK1, LeMAPKK3, and LeMAPKK4 clustered with the Ste7 subfamily (Figure 4F). Notably, no clear Mkk1/Mkk2 ortholog was detected, suggesting that the cell wall integrity pathway in *L. edodes* may rely on functional redundancy among multiple LeMAPKKs, a phenomenon well documented in other gene families [23,24], or that the canonical Mkk1/Mkk2 function has been replaced by a divergent or non-orthologous MAPKK in this species.

The three LeMAPKKKs could not be assigned to any yeast reference pathway (Ste11, Bck1, or Ssk2/22). Phylogenetic analysis revealed that they showed closer affinity to *A. thaliana* MAPKKKs than to fungal orthologs (Figure 5). This unexpected phylogenetic position may indicate lineage-specific functional diversification of MAPKKKs in *L. edodes*, possibly reflecting adaptive remodeling of its MAPK network driven by its wood-decaying ecological niche. Despite this ambiguity at the MAPKKK level, the clear subfamily assignments of LeMAPKs and LeMAPKKs allowed us to infer three potential MAPK signaling pathways in *L. edodes* (Figure 7): the pheromone/starvation pathway (LeMAPKKK1/2/3 → LeMAPKK1/3/4 → LeMAPK1/3/4/6/7/8), the hyperosmolarity pathway (LeMAPKKK1/2/3 → LeMAPKK2 → LeMAPK2), and the cell wall integrity pathway (LeMAPKKK1/2/3 → LeMAPKK? → LeMAPK5).

Several limitations of the identification strategy should be acknowledged. The approach relied primarily on the conserved kinase domain PF00069, and a single-domain search may fail to capture atypical family members with highly divergent or even lost canonical kinase domains; therefore, the possibility cannot be excluded that additional MAPK cascade-related genes exist in the *L. edodes* genome. Moreover, the phylogenetic classification of MAPKKKs remains challenging, as their sequence divergence makes confident assignment to specific subfamilies difficult. The inclusion of *A. thaliana* reference sequences helped to partially resolve these ambiguities (Figure 5), but the evolutionary distance between fungi and plants limits the interpretative power of such cross-kingdom comparisons. These limitations should be considered when interpreting the classification results presented in this study.

### 4.2. Strain-Specific, Dose-Dependent, and Temporally Dynamic Expression Under Cd Stress in L. edodes

The 16 MAPK genes exhibited three distinct regulatory patterns: strain specificity, dose dependence, and temporal dynamics.

Strain-specific patterns. The markedly faster and broader response in strain 4625 suggests that strain-specific genetic background determines the sensitivity of MAPK-mediated stress signaling. This is consistent with the mechanism observed in other fungi, where strain-level differences in regulatory networks, rather than gene presence/absence, are the primary drivers of differential stress responses. For instance, in *A. fumigatus*, high Cd tolerance in strain Af293 results from transcriptional variation in the efflux pump gene *pcaA* [25,26]; in *L. edodes*, differential expression of *LeAmy* correlates with Cd tolerance between strains [27]. Our data extend these observations to the MAPK cascade level, revealing that the entire signaling network—not just individual downstream effectors—is subject to strain-specific regulation.

Dose-dependent patterns. The concentration-dependent upregulation of LeMAPK6/7/8 and *LeMAPKK2*, with *LeMAPK7* behaving as a strain-independent sensor, suggests a quantitative decoding mechanism in which Cd dosage is translated into graded transcriptional outputs. The biphasic response of *LeMAPK5* (low-dose induction, high-dose repression) in strain 4625 indicates that distinct MAPK members employ fundamentally different response strategies: broad-range sensors (*LeMAPK7*) versus narrow-range or stress-threshold responders (*LeMAPK5*). This functional diversification aligns with the paradigm established in yeast, where low and high Cd concentrations activate qualitatively distinct pathways—oxidative stress responses at low doses and autophagy at high doses [28]. Our results suggest that this dose-dependent pathway selection is conserved in *L. edodes*, but with member-specific roles that may reflect adaptation to its ecological niche.

Temporal dynamics. The early response at 0.5 h in strain 4625, followed by a broader wave at 7 h, matches our previous multi-omics data [4,10]. Notably, Zhao et al. reported MAPK involvement at 24 h post-Cd exposure [29], indicating that MAPK regulation in *L. edodes* is not confined to the rapid phase but extends to sustained stress adaptation. Our 0.5–7 h window captures the initiation and amplification phases, providing a temporal framework for understanding how early signals are propagated to long-term responses.

Together, these results indicate that the MAPK cascade integrates strain-specific background, stress intensity, and exposure duration. *LeMAPK7* functions as a strain-independent concentration sensor, while *LeMAPK5* acts as a strain-specific threshold responder. This division of labor among MAPK members may enhance the network’s capacity to mount appropriate responses to diverse Cd stress conditions.

### 4.3. A Working Model for Cd-Activated MAPK Signaling in L. edodes

qRT-PCR analysis revealed that the three pathways inferred in Section 4.1, namely the pheromone/starvation pathway, the hyperosmolarity pathway, and the cell wall integrity pathway, all exhibited transcriptional responses to Cd stress, with their component genes showing differential expression across strains and concentrations (Figure 6). This indicates that Cd stress in *L. edodes* activates a multi-pathway cooperative response network rather than a single linear cascade. Notably, *LeMAPK1*, *LeMAPK7*, *LeMAPKK2*, and *LeMAPKKK1* were consistently upregulated in both strains at high Cd concentrations (1 and 10 mg/L) after 7 h of treatment. This strain-independent and robust activation suggests that these genes may constitute the broad-spectrum core components of the Cd-responsive MAPK network.

Beyond these core genes, the remaining genes exhibited strain-specific response patterns. For instance, in strain 4625, *LeMAPK6* and *LeMAPK8* were significantly upregulated at all three concentrations after 7 h, whereas in strain 4606, their responses were barely detectable, with significant upregulation observed only at 10 mg/L after 7 h. *LeMAPKK3* was significantly downregulated in strain 4625 under high-concentration (1 and 10 mg/L) treatment, but showed no obvious change in strain 4606. In addition, *LeMAPK5*, the sole Slt2-type MAPK (Figure 4C), displayed a biphasic response, induced at low Cd but repressed at high Cd, suggesting that the cell wall integrity pathway is activated only under mild stress and actively downregulated when stress intensifies, possibly reflecting a survival strategy that prioritizes core resources over cell wall maintenance under severe stress. *LeMAPK2*, the only Hog1-type MAPK (Figure 4D), showed little transcriptional response to Cd despite significant upregulation of its upstream kinase *LeMAPKK2* (Figure 6(B1–B2,K1–K2)). This uncoupling implies that *LeMAPK2* may primarily respond to osmotic stress rather than heavy metal stress [30], or that it may be activated via post-translational phosphorylation rather than transcriptional regulation. Taken together, these expression patterns suggest that the Cd-responsive MAPK network in *L. edodes* may operate under a complex regulatory architecture characterized by broad-spectrum core responses, strain-specific regulation, and pathway division of labor, and this transcriptional-level evidence provides preliminary support for understanding the operational logic of this network.

Functional redundancy and non-canonical activation. The absence of a clear Mkk1/Mkk2 ortholog in *L. edodes* (Figure 4E), together with the partial co-expression of LeMAPKK1/3/4 with downstream MAPKs, supports the involvement of functional redundancy among LeMAPKKs in signal transduction, a phenomenon documented in other organisms [23,24]. Furthermore, the unexpected phylogenetic affinity of LeMAPKKKs to plant homologs (Figure 5), combined with the lack of a complete canonical MAPKK complement, suggests that non-canonical activation mechanisms—such as MAPKK-independent phosphorylation—may operate in *L. edodes*. Notably, such non-canonical activation mechanisms have been reported not only in plants [31,32], but also in Saccharomyces cerevisiae, where hyperactive mutants of the Hog1 MAPK that are catalytically active without requiring MAPKK-mediated phosphorylation have been isolated [33]. In addition, under Cd stress, the Sty1 MAPK in fission yeast can be activated through an alternative pathway that does not depend on upstream MAPKKKs [34]. These examples demonstrate that non-canonical MAPK activation is possible across diverse fungal lineages. However, the existence of such mechanisms in *L. edodes* remains speculative and will require direct experimental validation through phosphorylation and protein–protein interaction assays.

Mycelial growth and phenotypic changes are important indicators for assessing Cd stress effects. Under long-term Cd treatment, phenotypic alterations are indeed detectable. Chen et al. observed that high Cd concentrations induced mycelial deformation and cell wall damage in *L. edodes* cultured on PDA plates containing high Cd concentrations for 7 days [5]. Consistently, our preliminary liquid culture experiments also showed that continuous Cd treatment for 7 days significantly inhibited mycelial biomass in a dose-dependent manner (0, 0.1, 1, 5, and 10 mg/L) [4]. However, differentially expressed genes detected under such long-term Cd treatment may largely reflect growth inhibition rather than Cd-specific stress responses, potentially interfering with the identification of key genes. Therefore, we adopted a short-term treatment strategy (0.5 h and 7 h) to capture early transcriptional responses to Cd stress. Within this time frame, we did not observe visible changes in mycelial pellet morphology or culture medium appearance; thus, we did not systematically quantify mycelial morphology or biomass.

Understanding the downstream targets of MAPK cascades is essential for elucidating how signals are transduced from perception to physiological adaptation. Under Cd stress, the biological significance of MAPK activation ultimately manifests as the regulation of effector proteins that directly execute cellular defense and detoxification functions. Downstream targets typically include transcription factors, transporters, and antioxidant enzymes. In fungi and plants, multiple MAPK downstream targets have been experimentally validated: In *S. pombe*, the transcription factor Atf1 is regulated by Sty1 MAPK through phosphorylation in response to Cd stress [34]. In rice, the metal transporter OsIRT1 interacts with OsMPK3 to participate in arsenic and iron stress responses, and the catalase OsCATA is phosphorylated by OsMAPK6 at Ser460 to regulate H_2_O_2_ homeostasis [35,36]. Collectively, these examples indicate that MAPK-mediated regulation of transcription factors, transporters, and antioxidant enzymes represents a conserved mechanism in eukaryotic responses to Cd stress. Notably, our previous transcriptomic data also identified multiple Cd-responsive genes belonging to the above categories, suggesting that they may serve as potential downstream targets of MAPK signaling in *L. edodes*, providing candidate targets for subsequent functional screening.

Since the proposed working model of MAPK-mediated Cd stress response in *L. edodes* (Section 4.1; Figure 7) is constructed based on phylogenetic inference and qRT-PCR data, this predictive model alone does not constitute a complete mechanistic explanation and must be tested and confirmed through experimental validation. MAPK cascades are inherently signal networks composed of multi-tiered cascades and cross-regulations, and their functional dissection requires iterative refinement between model prediction and experimental validation. Therefore, we selected *LeMAPK7* as an entry point to prioritize the dissection of its upstream and downstream signaling components, thereby progressively elucidating the MAPK signaling network in Cd stress response. At present, overexpression and knockdown constructs of *LeMAPK7* have been generated and are undergoing transformation and phenotypic evaluation in *L. edodes*. Upon establishment of transgenic lines, systematic identification of the upstream MAPKK and downstream interacting partners of *LeMAPK7* will be performed through gene expression analysis, yeast two-hybrid, GST pull-down, and BiFC assays, aiming to progressively delineate the key pathways mediated by *LeMAPK7* in Cd stress response. Since the above functional validation is still in progress, detailed results will be reported separately in future studies.

## Figures and Tables

**Figure 1:**
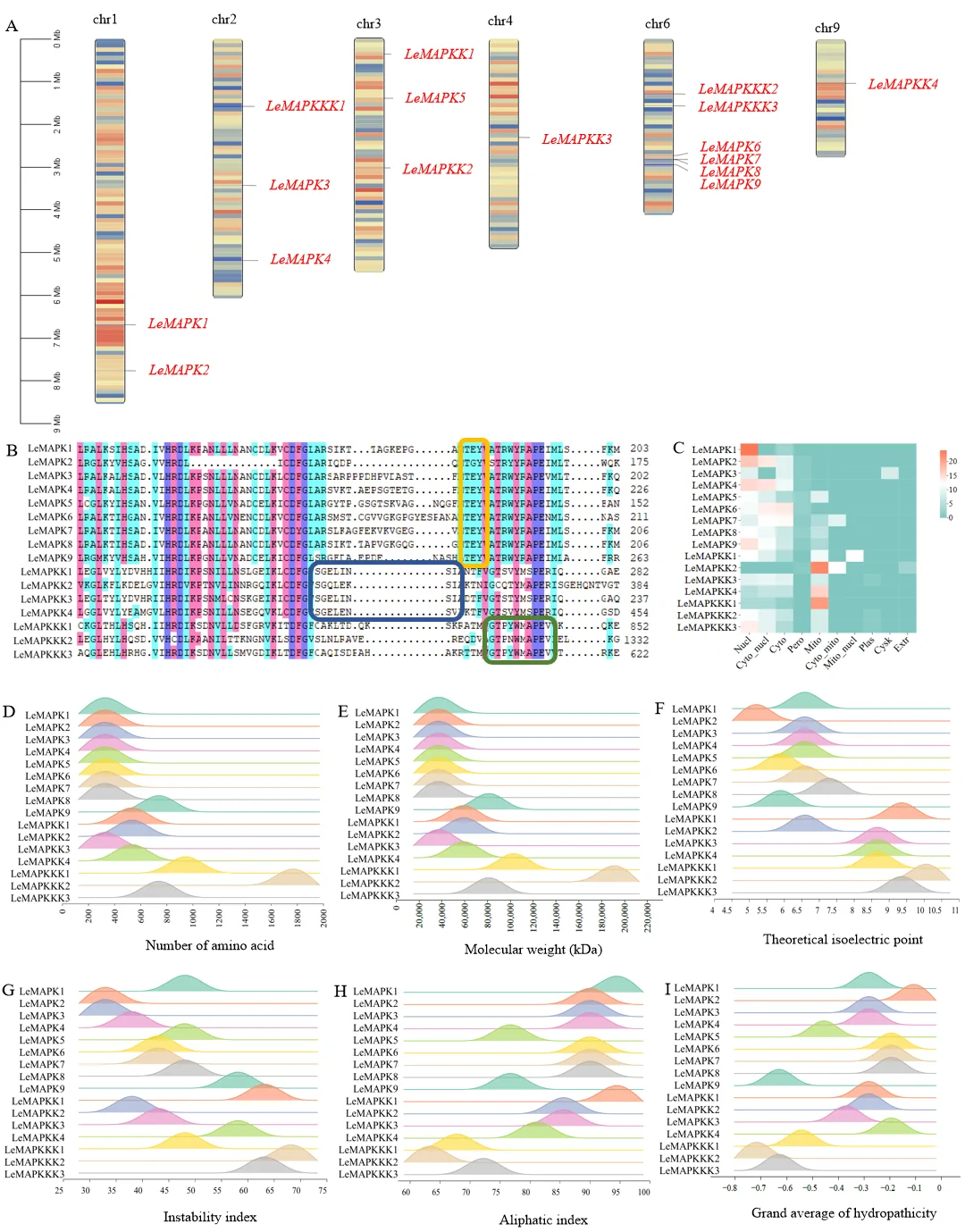
Chromosomal distribution, protein features, and physicochemical properties of the MAPK, MAPKK, and MAPKKK family members in *L. edodes*. (**A**) Chromosomal distribution of *LeMAPK*, *LeMAPKK*, and *LeMAPKKK* genes on the 10 chromosomes of *L. edodes.* The scale bar represents chromosome length in megabases (Mb). Chromosomal locations were extracted and visualized using TBtools-II (v2.458). (**B**) Family-specific conserved motifs of the protein sequences. The family-specific conserved motifs are as follows: Yellow, TXY motif of MAPKs; Blue, S/TXXXXXS/T motif of MAPKKs; Green, -GTXX(W/Y)MAPE-, -GTPEFMAPE(L/V/M)(Y/F/L)-, or -G(T/S)PX(F/Y/W)MAPEV- motifs of MAPKKKs. (**C**) Subcellular localization predictions, as predicted by WoLF PSORT using the “fungi” organism option. The heatmap shows the predicted localization probabilities across eight compartments: nucleus (Nucl), cytoplasm (Cyto), mitochondria (Mito), plasma membrane (Plas), cytoskeleton (Cysk), extracellular space (Extr), peroxisome (Pero), and others. (**D**–**I**) Physicochemical properties of the three protein families, as predicted using the Protein Parameter Calc tool in TBtools-II (v2.458): (**D**) number of amino acids (protein length), (**E**) molecular weight (kDa), (**F**) theoretical isoelectric point (pI), (**G**) instability index, (**H**) aliphatic index, and (**I**) grand average of hydropathicity (GRAVY).

**Figure 2:**
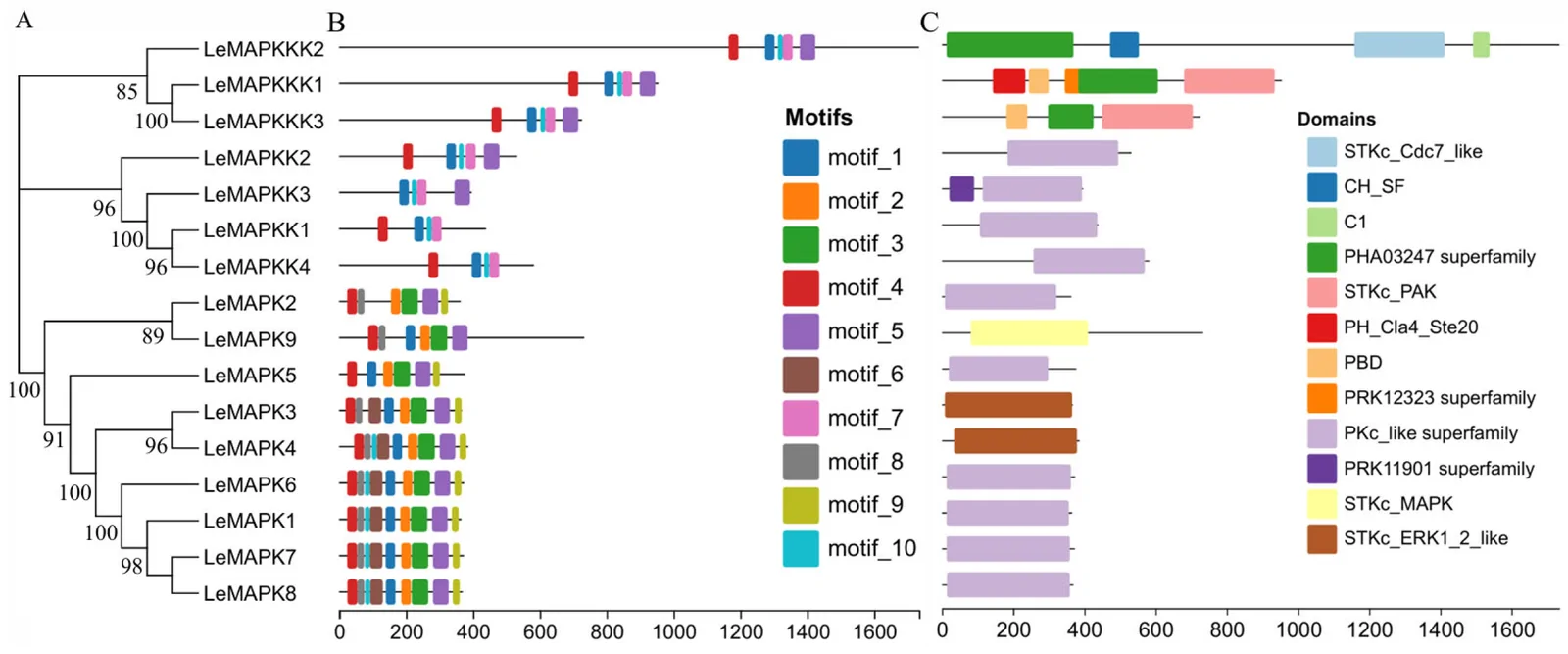
Phylogenetic relationships, conserved motifs, and conserved domains of the MAPK, MAPKK, and MAPKKK family members in *L. edodes*. (**A**) Phylogenetic trees, constructed using the neighbor-joining (NJ) method in MEGA 11. Numbers at the nodes indicate bootstrap support values (%) from 1000 replicates. (**B**) Conserved motif compositions, as predicted by the MEME suite with the number of motifs set to 10. Each conserved motif is represented by a distinct colored box. The scale bar indicates amino acid length. (**C**) Conserved domain architectures, as validated by the NCBI Batch CD-Search tool. The domains are represented by colored boxes, with different colors indicating different domain types. The scale bar indicates amino acid length.

**Figure 3:**
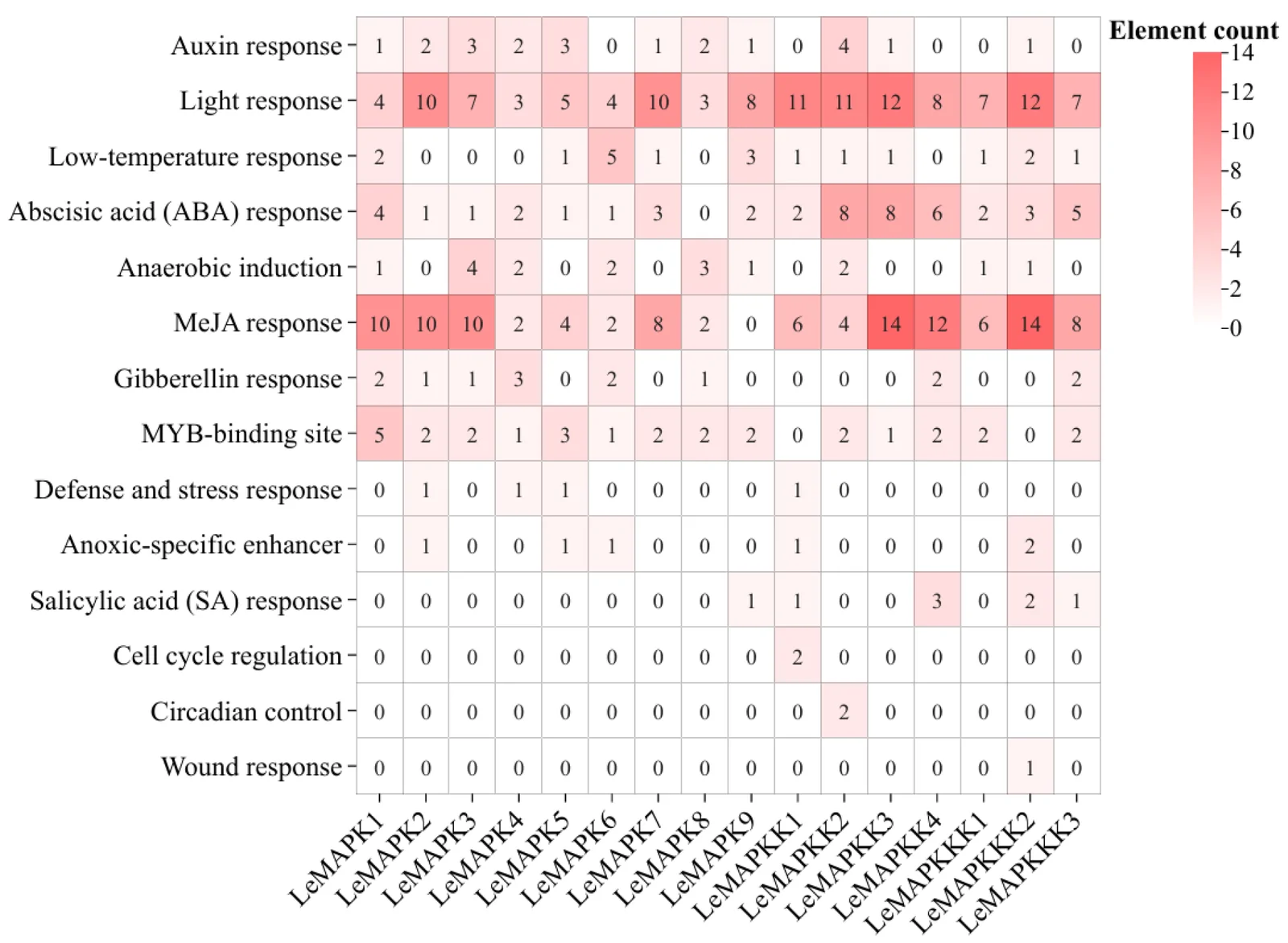
Characterization of promoter *cis*-elements in *LeMAPK*, *LeMAPKK*, and *LeMAPKKK* genes. The heatmap shows the number of *cis*-elements per category across the three gene families, as predicted by the PlantCARE database. Raw prediction results were filtered to remove redundant information.

**Figure 4:**
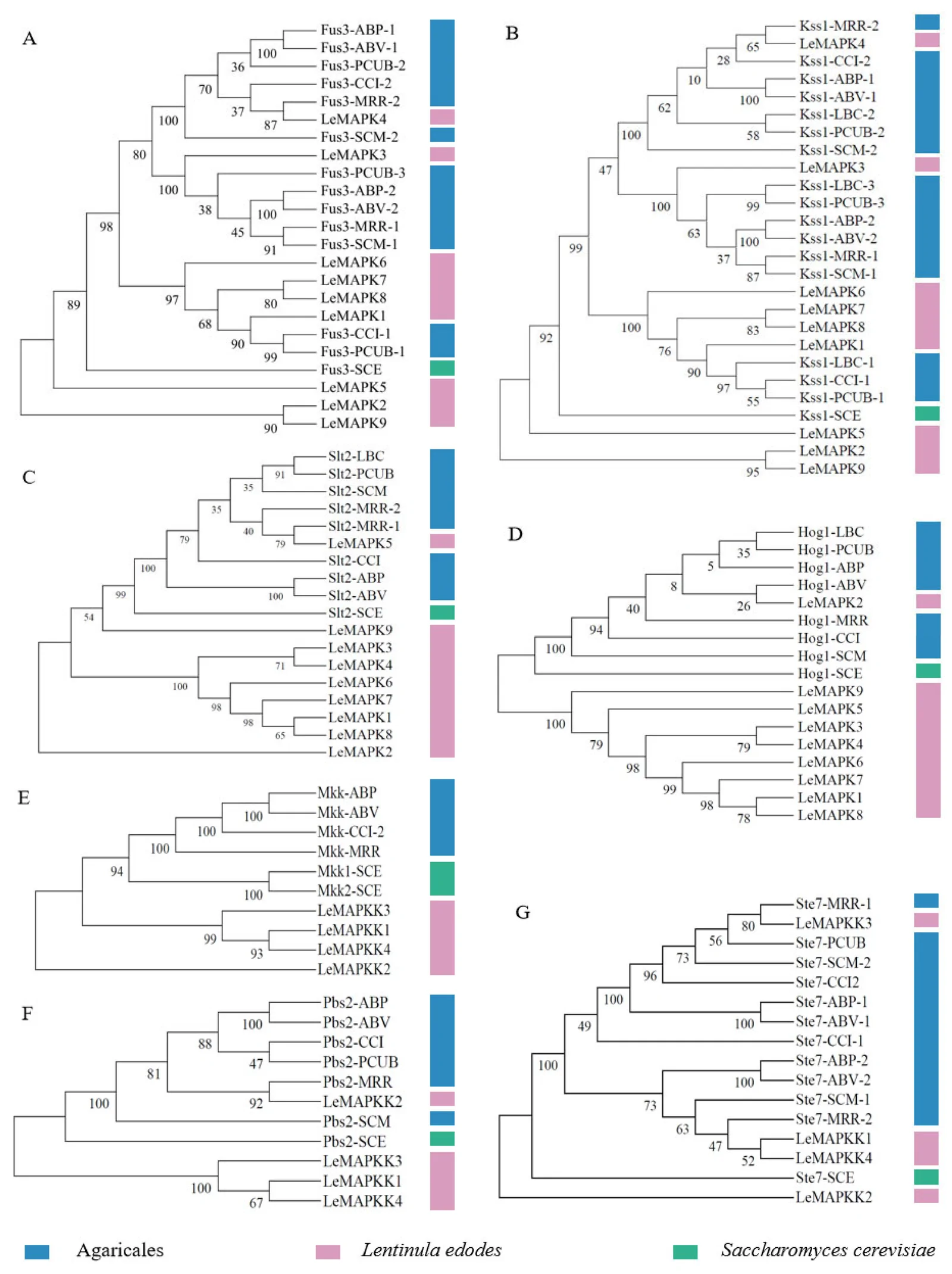
Phylogenetic and homology analysis of LeMAPKs and LeMAPKKs across species of Agaricales and *Saccharomyces cerevisiae*. (**A**–**D**) Phylogenetic relationships of the nine LeMAPKs with homologs from Agaricales species and *S. cerevisiae*. The trees show the clustering patterns of LeMAPKs with Fus3 homologs (**A**), Kss1 homologs (**B**), Slt2 homologs (**C**), and Hog1 homologs (**D**). (**E**–**G**) Phylogenetic relationships of the four LeMAPKKs with homologs from Agaricales species and *S. cerevisiae*. The trees show the clustering patterns of LeMAPKKs with Mkk homologs (**E**), Ste7 homologs (**F**), and Pbs2 homologs (**G**). *L. edodes* sequences (LeMAPKs and LeMAPKKs) are highlighted in pink, Agaricales homologs are shown in blue, and *S. cerevisiae* homologs are shown in green. Reference sequences for each group were retrieved from the KEGG database, and the phylogenetic trees were constructed using MEGA 11 with the NJ method. Numbers at the nodes indicate bootstrap support values (%) from 1000 replicates.

**Figure 5:**
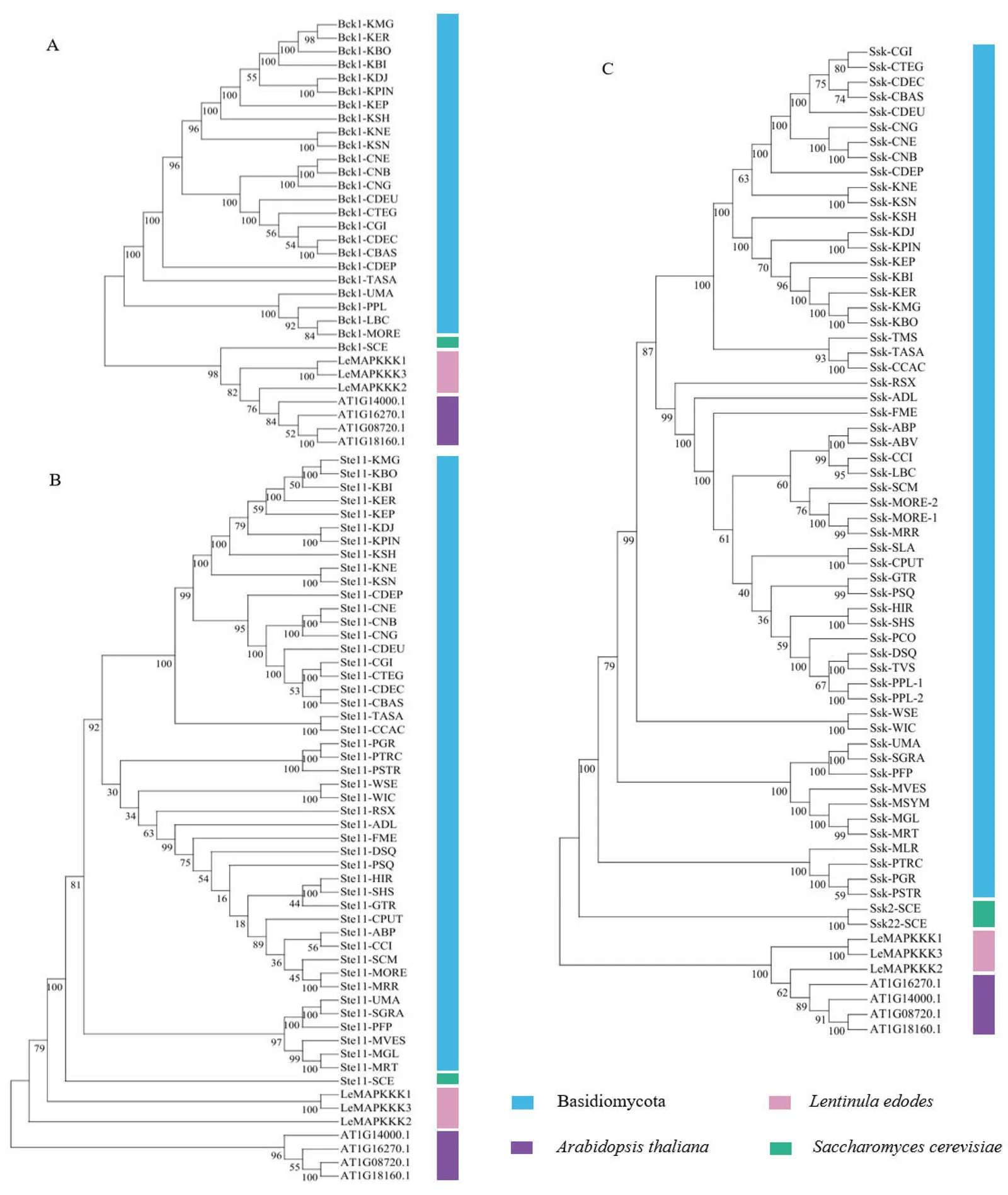
Phylogenetic and homology analysis of LeMAPKKKs across representative species of Basidiomycota, *S. cerevisiae*, and *A. thaliana*. The trees show the clustering patterns of LeMAPKKKs with Bck1 homologs (**A**), Ste11 homologs (**B**), and Ssk homologs (**C**). *L. edodes* sequences (LeMAPKKKs) are highlighted in pink, Basidiomycota homologs are shown in blue, *S. cerevisiae* homologs are shown in green, and *A. thaliana* homologs are shown in purple. Reference sequences for each group were retrieved from the KEGG database, and *A. thaliana* sequences were obtained from The Arabidopsis Information Resource (TAIR). Phylogenetic trees were constructed using MEGA 11 with the NJ method. Numbers at the nodes indicate bootstrap support values (%) from 1000 replicates.

**Figure 6:**
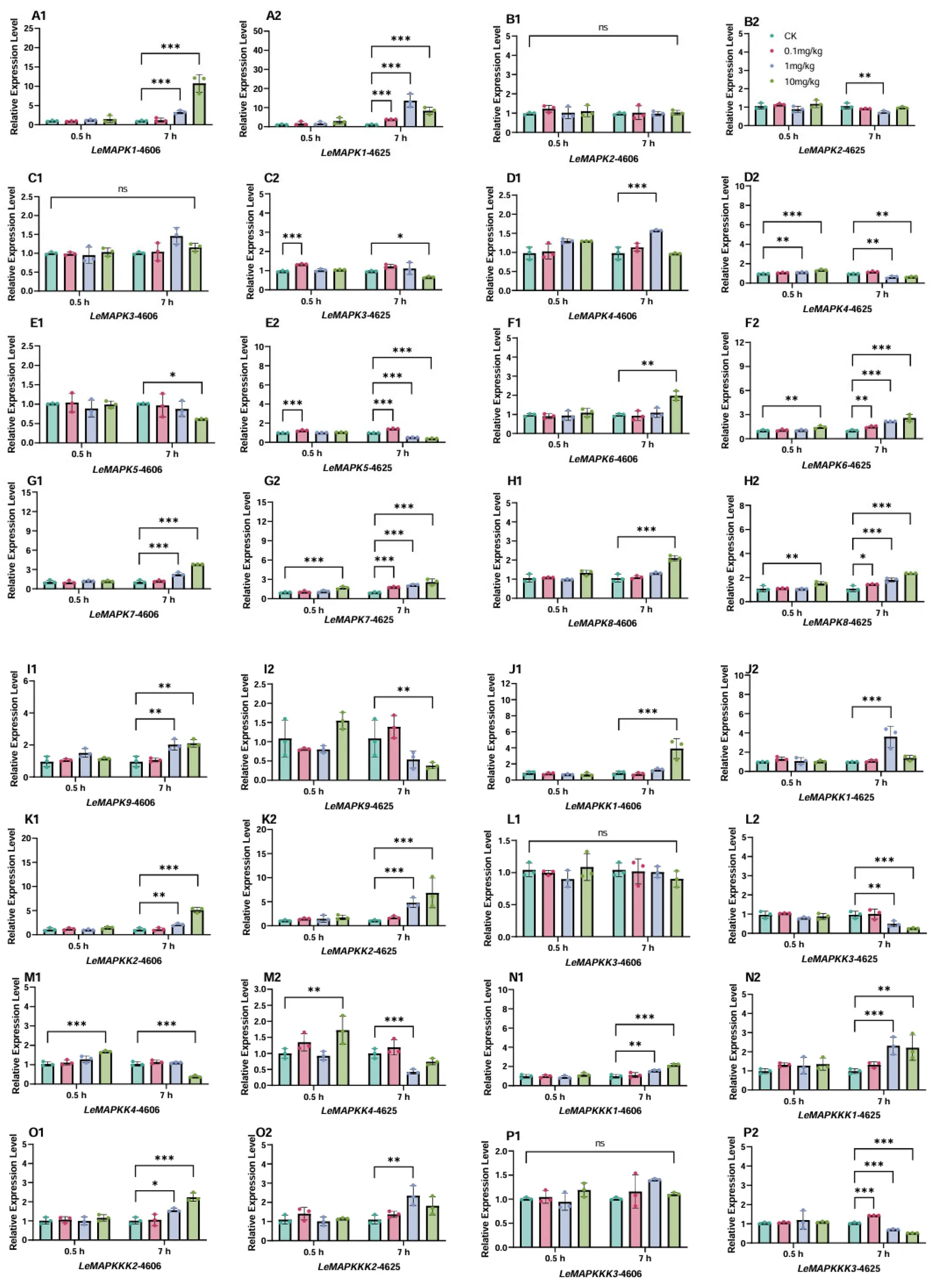
Cd-induced expression patterns of *LeMAPK*, *LeMAPKK*, and *LeMAPKKK* genes. qRT-PCR analysis was performed to examine the transcriptional responses of nine *LeMAPK* genes, four *LeMAPKK* genes, and three *LeMAPKKK* genes in *L. edodes* strains 4606 (low Cd-accumulating) and 4625 (high Cd-accumulating) under CdCl_2_ stress at two time points (0.5 h and 7 h) and three concentrations (0.1, 1, and 10 mg/L). The two strains were selected for their contrasting Cd accumulation capacities, and the concentrations and time points were chosen based on preliminary experiments showing significant differences in mycelial biomass, enzymatic activities, Cd accumulation, and antioxidant responses under these conditions. (Panels **A1**–**P1**) show the expression profiles of strain 4606, and (Panels **A2**–**P2**) show those of strain 4625, with each panel representing one gene ((**A1**–**I1**,**A2**–**I2**) LeMAPK1–9; (**J1**–**M1**,**J2**–**M2**) LeMAPKK1–4; (**N1**–**P1**,**N2**–**P2**) LeMAPKKK1–3). Relative expression levels were calculated using the 2^−ΔΔCt^ method, with the *L. edodes Actin* gene as an internal reference and untreated strains as controls. Data are presented as fold change relative to the control (0 mg/L) at each time point. Each sample included three biological replicates and three technical replicates. Error bars represent standard deviation (SD). Statistical significance was determined using one-way ANOVA followed by the Benjamini–Hochberg false discovery rate (FDR) procedure for multiple-comparison correction. *, **, and *** indicate adjusted *p*-values (q-value) < 0.05, <0.01, and <0.001, respectively; ns, not significant.

**Figure 7:**
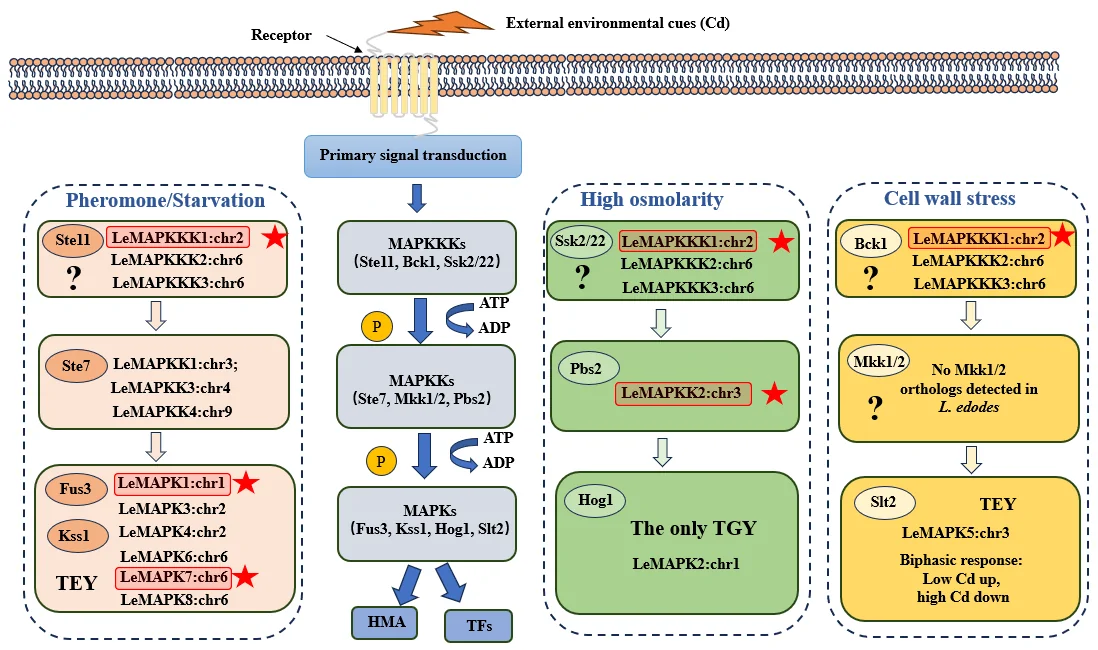
Proposed working model of Cd-activated MAPK signaling pathways in *L. edodes*. Three pathways are inferred: the pheromone/starvation pathway (LeMAPKKK1/2/3 → LeMAPKK1/3/4 → LeMAPK1/3/4/6/7/8), the hyperosmolarity pathway (LeMAPKKK1/2/3 → LeMAPKK2 → LeMAPK2), and the cell wall integrity pathway (LeMAPKKK1/2/3 → LeMAPKK? → LeMAPK5). *LeMAPK1*, *LeMAPK7*, *LeMAPKK2*, and *LeMAPKKK1* are identified as the broad-spectrum core components of the Cd-responsive MAPK network, highlighted with red five-pointed stars. The remaining members exhibit strain-specific or specialized regulatory responses. Solid arrows denote phosphorylation and activation. Question marks indicate uncertainty due to the absence of identified homologs at the corresponding levels. This model, constructed based on phylogenetic and qRT-PCR data, requires experimental validation through protein–protein interaction assays and mutant phenotyping in future studies.

## Data Availability

The original contributions presented in this study are included in the article/Supplementary Material. Further inquiries can be directed to the corresponding authors.

## Supplementary Materials

The following supporting information can be downloaded at: https://www.mdpi.com/article/10.3390/jof12100713/s1, Table S1. List of identified MAPK cascade genes and their corresponding accession numbers in *L. edodes*; Table S2. List of fungal species and corresponding sequence identifiers used as references for phylogenetic analysis of MAPK gene families; Table S3. List of fungal species and corresponding sequence identifiers used as references for phylogenetic analysis of MAPKK gene families; Table S4. List of fungal species and corresponding sequence identifiers used as references for phylogenetic analysis of MAPKKK gene families; Table S5. Sequence identity between LeMAPKKKs and their yeast homologs (Ssk, Bck1, and Ste11); Figure S1. Phylogenetic and homology analysis of LeMAPKKKs in *L. edodes* and *Arabidopsis thaliana*. The phylogenetic tree shows the clustering patterns of two *L. edodes* LeMAPKKKs with MAPKKK homologs in *A. thaliana*. *L. edodes* sequences (LeMAPKKKs) are highlighted in pink, and *A. thaliana* homologs are shown in purple. *A. thaliana* sequences were obtained from The Arabidopsis Information Resource (TAIR). The phylogenetic tree was constructed using MEGA 11 with the NJ method.
