# Supplementary material for: Deciphering the Molecular Basis of Cadmium Stress Adaptation in Lentinula edodes: Genome-Wide Identification of MAPK Cascade Genes and Their Functional Characterization
Source: J Fungi (Basel). 2026 Sep 23;12(10):713. doi: 10.3390/jof12100713 (PMC13706460; doi:10.3390/jof12100713)
Supplement: Supplementary file 1 [file jof-12-00713-s001.zip › jof-4464603-supplementary.pdf]

Supplementary Data

Deciphering the Molecular Basis of Cadmium Stress Adaptation in *Lentinula edodes*: Genome-Wide Identification of MAPK Cascade Genes and Their Functional Characterization

Table S1. List of identified MAPK cascade genes and their corresponding accession numbers in *L. edodes*

Gene designation	Protein ID	Gene ID
LeMAPK1	KAF8832462.1	HHX47_DHR1001485
LeMAPK2	KAF8832774.1	HHX47_DHR1002005
LeMAPK3	KAF8830351.1	HHX47_DHR2000497
LeMAPK4	KAF8830710.1	HHX47_DHR2001002
LeMAPK5	KAF8828761.1	HHX47_DHR3000059
LeMAPK6	KAF8825958.1	HHX47_DHR6000732
LeMAPK7	KAF8825980.1	HHX47_DHR6000391
LeMAPK8	KAF8825983.1	HHX47_DHR6000456
LeMAPK9	KAF8825993.1	HHX47_DHR6000464
LeMAPKK1	KAF8828522.1	HHX47_DHR3000096
LeMAPKK2	KAF8829109.1	HHX47_DHR3001107
LeMAPKK3	KAF8827911.1	HHX47_DHR4000892
LeMAPKK4	KAF8823805.1	HHX47_DHR9000396
LeMAPKKK1	KAF8829983.1	HHX47_DHR2000245
LeMAPKKK2	KAF8825708.1	HHX47_DHR6000200
LeMAPKKK3	KAF8825744.1	HHX47_DHR6000045

Table S2. List of fungal species and corresponding sequence identifiers used as references for phylogenetic analysis of MAPK gene families

Custom ID	Species	Entry ID	NCBI-ProteinID:
Fus3-ABP-1	*Agaricus bisporus* var. burnettii JB137-S8	AGABI1DRAFT115254	XP_007331974
Fus3-ABP-2	*Agaricus bisporus* var. burnettii JB137-S8	AGABI1DRAFT86785	XP_007332224
Fus3-ABV-1	*Agaricus bisporus* var. bisporus H97	AGABI2DRAFT194216	XP_006463375
Fus3-ABV-2	*Agaricus bisporus* var. bisporus H97	AGABI2DRAFT195979	XP_006457118
Fus3-CCI-1	*Coprinopsis cinerea* (gray shag)	CC1G_03633	XP_001828839
Fus3-CCI-2	*Coprinopsis cinerea* (gray shag)	CC1G_07620	XP_001832360
Fus3-MRR-1	*Moniliophthora roreri*	Moror_16192	ESK89417
Fus3-MRR-2	*Moniliophthora roreri*	Moror_7769	ESK90147
Fus3-PCUB-1	*Psilocybe cubensis* (magic mushroom)	JR316_0000861	XP_047754421
Fus3-PCUB-2	*Psilocybe cubensis* (magic mushroom)	JR316_0008886	XP_047746056
Fus3-PCUB-3	*Psilocybe cubensis* (magic mushroom)	JR316_0009268	XP_047746432
Fus3-SCE	*Saccharomyces cerevisiae* (budding yeast)	YBL016W	NP_009537
Fus3-SCM-1	*Schizophyllum commune*	SCHCO_02636914	XP_003028977
Fus3-SCM-2	*Schizophyllum commune*	SCHCO_02641010	XP_050197988
Slt2-ABP	*Agaricus bisporus* var. burnettii JB137-S8	AGABI1DRAFT112477	XP_007328348
Slt2-ABV	*Agaricus bisporus* var. bisporus H97	AGABI2DRAFT192446	XP_006460856
Slt2-CCI	*Coprinopsis cinerea* (gray shag)	CC1G_02007	XP_001830371
Slt2-LBC	*Laccaria bicolor*	LACBIDRAFT_232153	XP_001877809
Slt2-MRR-1	*Moniliophthora roreri*	Moror_1513	ESK93696

Slt2-MRR-2	*Moniliophthora roreri*	Moror_1769	ESK93358
Slt2-PCUB	*Psilocybe cubensis* (magic mushroom)	JR316_0002066	XP_047752784
Slt2-SCM	*Schizophyllum commune*	SCHCO_02631606	XP_003030159
Slt2-SCE	*Saccharomyces cerevisiae* (budding yeast)	YHR030C	NP_011895
Hog1-SCE	*Saccharomyces cerevisiae* (budding yeast)	YLR113W	NP_013214
Hog1-ABP	*Agaricus bisporus* var. burnettii JB137-S8	AGABI1DRAFT_22411	XP_007324914
Hog1-ABV	*Agaricus bisporus* var. bisporus H97	AGABI2DRAFT41003	XP_006453743
Hog1-CCI	*Coprinopsis cinerea* (gray shag)	CC1G_00577	XP_001829398
Hog1-LBC	*Laccaria bicolor*	LACBIDRAFT_187188	XP_001873516
Hog1-MRR	*Moniliophthora roreri*	Moror_7279	ESK96138
Hog1-PCUB	*Psilocybe cubensis* (magic mushroom)	JR316_0000514	XP_047754074
Hog1-SCM	*Schizophyllum commune*	SCHCO_02175896	XP_003037016
Kss1-SCE	*Saccharomyces cerevisiae* (budding yeast)	YGR040W	NP_011554
Kss1-ABP-1	*Agaricus bisporus* var. burnettii JB137-S8	AGABI1DRAFT115254	XP_007331974
Kss1-ABP-2	*Agaricus bisporus* var. burnettii JB137-S8	AGABI1DRAFT86785	XP_007332224
Kss1-ABV-1	*Agaricus bisporus* var. bisporus H97	AGABI2DRAFT194216	XP_006463375
Kss1-ABV-2	*Agaricus bisporus* var. bisporus H97	AGABI2DRAFT195979	XP_006457118
Kss1-CCI-1	*Coprinopsis cinerea* (gray shag)	CC1G_03633	XP_001828839
Kss1-CCI-2	*Coprinopsis cinerea* (gray shag)	CC1G_07620	XP_001832360
Kss1-LBC-1	*Laccaria bicolor*	LACBIDRAFT_174940	XP_001886883
Kss1-LBC-2	*Laccaria bicolor*	LACBIDRAFT_184962	XP_001881046
Kss1-LBC-3	*Laccaria bicolor*	LACBIDRAFT_250005	XP_001881172
Kss1-MRR-1	*Moniliophthora roreri*	Moror_16192	ESK89417
Kss1-MRR-2	*Moniliophthora roreri*	Moror_7769	ESK90147
Kss1-PCUB-1	*Psilocybe cubensis* (magic mushroom)	JR316_0000861	XP_047754421
Kss1-PCUB-2	*Psilocybe cubensis* (magic mushroom)	JR316_0008886	XP_047746056
Kss1-PCUB-3	*Psilocybe cubensis* (magic mushroom)	JR316_0009268	XP_047746432
Kss1-SCM-1	*Schizophyllum commune*	SCHCO_02636914	XP_003028977
Kss1-SCM-2	*Schizophyllum commune*	SCHCO_02641010	XP_050197988

Table S3. List of fungal species and corresponding sequence identifiers used as references for phylogenetic analysis of MAPKK gene families

Custom ID	Species	Entry ID	NCBI-ProteinID:
Ste7-ABP-1	*Agaricus bisporus* var. burnettii JB137-S8	AGABI1DRAFT114072	XP_007330171
Ste7-ABP-2	*Agaricus bisporus* var. burnettii JB137-S8	AGABI1DRAFT123361	XP_007334254
Ste7-ABV-1	*Agaricus bisporus* var. bisporus H97	AGABI2DRAFT186680	XP_006462828
Ste7-ABV-2	*Agaricus bisporus* var. bisporus H97	AGABI2DRAFT187496	XP_006464165
Ste7-CCI-1	*Coprinopsis cinerea* (gray shag)	CC1G_02205	XP_001834469
Ste7-CCI2	*Coprinopsis cinerea* (gray shag)	CC1G_03368	XP_001835586
Ste7-PCUB	*Psilocybe cubensis* (magic mushroom)	JR316_0007318	XP_047748343
Ste7-SCM-1	*Schizophyllum commune*	SCHCO_02488207	XP_003038582
Ste7-SCM-2	*Schizophyllum commune*	SCHCO_02745977	XP_003034071
Ste7-SCE	*Saccharomyces cerevisiae* (budding yeast)	YDL159W	NP_010122
Ste7-MRR-1	*Moniliophthora roreri*	Moror_10814	ESK88009
Ste7-MRR-2	*Moniliophthora roreri*	Moror_16599	ESK86281
Mkk1-SCE	*Saccharomyces cerevisiae* (budding yeast)	YOR231W	NP_014874
Mkk2-SCE	*Saccharomyces cerevisiae* (budding yeast)	YPL140C	NP_015185
Mkk-ABP	*Agaricus bisporus* var. burnettii JB137-S8	AGABI1DRAFT38036	XP_007328046
Mkk-ABV	*Agaricus bisporus* var. bisporus H97	AGABI2DRAFT70301	XP_006460836
Mkk-CCI-2	*Coprinopsis cinerea* (gray shag)	CC1G_01983	XP_001830347
Mkk-MRR	*Moniliophthora roreri*	Moror_6820	ESK96600

Pbs2-SCE	*Saccharomyces cerevisiae* (budding yeast)	YJL128C	NP_012407
Pbs2-ABP	*Agaricus bisporus* var. burnettii JB137-S8	AGABI1DRAFT71099	XP_007328123
Pbs2-ABV	*Agaricus bisporus* var. bisporus H97	AGABI2DRAFT185122	XP_006460780
Pbs2-CCI	*Coprinopsis cinerea* (gray shag)	CC1G_14241	XP_002911710
Pbs2-MRR	*Moniliophthora roreri*	Moror_6763	ESK96543
Pbs2-PCUB	*Psilocybe cubensis* (magic mushroom)	JR316_0002011	XP_047752729
Pbs2-SCM	*Schizophyllum commune*	SCHCO_02669819	XP_003030014

Table S4. List of fungal species and corresponding sequence identifiers used as references for phylogenetic analysis of MAPKKK gene families

Custom ID	Species	Entry ID	NCBI-ProteinID:
Ste11-ABP	*Agaricus bisporus* var. burnettii JB137-S8	AGABI1DRAFT68456	XP_007326241
Ste11-CCI	*Coprinopsis cinerea* (gray shag)	CC1G_12014	XP_001833189
Ste11-MORE	*Marasmius oreades* (fairy-ring mushroom)	E1B28_008848	XP_043008967
Ste11-MRR	*Moniliophthora roreri*	Moror_6600	ESK97022
Ste11-SCE	*Saccharomyces cerevisiae* (budding yeast)	YLR362W	NP_013466
Ste11-SCM	*Schizophyllum commune*	SCHCO_02605129	XP_050202733
Ste11-CPUT	*Coniophora puteana*	CONPUDRAFT_87650	XP_007764731
Ste11-RSX	*Rhizoctonia solani*	RhiXN_01553	XP_043187195
Ste11-GTR	*Gloeophyllum trabeum*	GLOTRDRAFT_72507	XP_007863496
Ste11-FME	*Fomitiporia mediterranea*	FOMMEDRAFT_157522	XP_007267720
Ste11-ADL	*Auricularia subglabra*	AURDEDRAFT_81195	EJD53011
Ste11-PSQ	*Punctularia strigosozonata*	PUNSTDRAFT_114782	XP_007385611
Ste11-HIR	*Heterobasidion irregulare*	HETIRDRAFT_43369	XP_009541778
Ste11-SHS	*Stereum hirsutum*	STEHIDRAFT_153901	XP_007301103
Ste11-DSQ	*Dichomitus squalens*	DICSQDRAFT_77854	XP_007361196
Ste11-CNE	*Cryptococcus deneoformans* JEC21	CND05760	XP_570496
Ste11-CNB	*Cryptococcus deneoformans* B-3501A	CNBD0630	XP_776014
Ste11-CNG	*Cryptococcus neoformans* var. grubii H99	CNAG_06980	XP_012049620
Ste11-CGI	*Cryptococcus gattii*	CGB_I1040C	XP_003196109
Ste11-CDEU	*Cryptococcus deuterogattii*	CNBG_9621	XP_062886010
Ste11-CDEC	*Cryptococcus decagattii*	89991914	XP_064723026
Ste11-CTEG	*Cryptococcus tetragattii*	91992265	XP_066611811
Ste11-CBAS	*Cryptococcus bacillisporus*	91983632	XP_066607512
Ste11-CDEP	*Cryptococcus depauperatus*	91089620	XP_066070875
Ste11-KMG	*Kwoniella mangroviensis*	30162086	XP_019005420
Ste11-KNE	*Kwoniella newhampshirensis*	92183083	XP_066800688
Ste11-KDJ	*Kwoniella dejecticola*	28965825	XP_018265753
Ste11-KBI	*Kwoniella bestiolae*	30208477	XP_019047465
Ste11-KPIN	*Kwoniella pini*	30174807	XP_019008756
Ste11-KSH	*Kwoniella shivajii*	87954097	XP_062789765
Ste11-KER	*Kwoniella europaea*	91104857	XP_066085915
Ste11-KSN	*Kwoniella shandongensis*	43588018	XP_031861889
Ste11-KBO	*Kwoniella botswanensis*	90018173	XP_064748956
Ste11-KEP	*Kwoniella dendrophila*	91092786	XP_066073989
Ste11-TASA	*Trichosporon asahii* var. asahii	A1Q1_07596	XP_014182083
Ste11-CCAC	*Cutaneotrichosporon cavernicola*	CcaHIS019_0307930	XP_060455988
Ste11-WSE	*Wallemia mellicola*	WALSEDRAFT_17415	XP_006957738
Ste11-WIC	*Wallemia ichthyophaga*	J056_001541	XP_009269444

Ste11-UMA	*Mycosarcoma maydis*	UMAG_04258	XP_011390727
Ste11-PFP	*Pseudozyma flocculosa*	PFL1_06027	XP_007881756
Ste11-SGRA	*Sporisorium graminicola*	EX895_002006	XP_029741460
Ste11-MGL	*Malassezia globosa*	MGL_3142	XP_001729598
Ste11-MRT	*Malassezia restricta*	MRET_1335	XP_027483558
Ste11-MVES	*Malassezia vespertilionis*	80903454	XP_056064728
Ste11-PGR	*Puccinia graminis*	PGTG_08104	XP_003326274
Ste11-PTRC	*Puccinia triticina* (wheat leaf rust)	PtA15_10A243	XP_053024378
Ste11-PSTR	*Puccinia striiformis* f. sp. tritici	Pst134EA_019249	XP_047803170
Ssk2-SCE	*Saccharomyces cerevisiae* (budding yeast)	YNR031C	NP_014428
Ssk22-SCE	*Saccharomyces cerevisiae* (budding yeast)	YCR073C	NP_009998
Ssk-ABP	*Agaricus bisporus* var. burnettii JB137-S8	AGABI1DRAFT117102	XP_007325488
Ssk-ABV	*Agaricus bisporus* var. bisporus H97	AGABI2DRAFT214884	XP_006454607
Ssk-CCI	*Coprinopsis cinerea* (gray shag)	CC1G_13687	XP_002912155
Ssk-LBC	*Laccaria bicolor*	LACBIDRAFT_141037	XP_001873326
Ssk-MORE-1	*Marasmius oreades* (fairy-ring mushroom)	E1B28_001410	XP_043016050
Ssk-MORE-2	*Marasmius oreades* (fairy-ring mushroom)	E1B28_010501	XP_043007940
Ssk-MRR	*Moniliophthora roreri*	Moror_310	ESK98193
Ssk-SCM	*Schizophyllum commune*	SCHCO_02488497	XP_050202197
Ssk-SLA	*Serpula lacrymans*	SERLADRAFT_413324	XP_007314614
Ssk-CPUT	*Coniophora puteana*	CONPUDRAFT_141011	XP_007763268
Ssk-RSX	*Rhizoctonia solani*	RhiXN_01750	XP_043187392
Ssk-GTR	*Gloeophyllum trabeum*	GLOTRDRAFT_53823	XP_007860421
Ssk-FME	*Fomitiporia mediterranea*	FOMMEDRAFT_28004	XP_007265862
Ssk-ADL	*Auricularia subglabra*	AURDEDRAFT_132470	EJD55307
Ssk-PSQ	*Punctularia strigosozonata*	PUNSTDRAFT_48388	XP_007378323
Ssk-HIR	*Heterobasidion irregulare*	HETIRDRAFT_167185	XP_009541498
Ssk-SHS	*Stereum hirsutum*	STEHIDRAFT_89904	XP_007298329
Ssk-PCO	*Phanerochaete carnosa*	PHACADRAFT_179988	XP_007390256
Ssk-DSQ	*Dichomitus squalens*	DICSQDRAFT_46396	XP_007359834
Ssk-TVS	*Trametes versicolor*	TRAVEDRAFT_55421	XP_008032381
Ssk-PPL-1	*Postia placenta*	POSPLDRAFT_50885	EED81252
Ssk-PPL-2	*Postia placenta*	POSPLDRAFT_90009	EED81516
Ssk-CNE	*Cryptococcus deneoformans* JEC21	CNL05560	XP_568092
Ssk-CNB	*Cryptococcus deneoformans* B-3501A	CNBI1260	XP_773512
Ssk-CNG	*Cryptococcus neoformans* var. grubii H99	CNAG_05063	XP_012048481
Ssk-CGI	*Cryptococcus gattii*	CGB_D1460C	XP_003193371
Ssk-CDEU	*Cryptococcus deuterogattii*	CNBG_4621	XP_062884504
Ssk-CDEC	*Cryptococcus decagattii*	89987482	XP_064718662
Ssk-CTEG	*Cryptococcus tetragattii*	91986996	XP_066616616
Ssk-CBAS	*Cryptococcus bacillisporus*	91980763	XP_066604685
Ssk-CDEP	*Cryptococcus depauperatus*	91086918	XP_066068224
Ssk-KMG	*Kwoniella mangroviensis*	30165827	XP_019001309
Ssk-KNE	*Kwoniella newhampshirensis*	92181272	XP_066802496
Ssk-KDJ	*Kwoniella dejecticola*	28970431	XP_018261015
Ssk-KBI	*Kwoniella bestiolae*	30211701	XP_019044022
Ssk-KPIN	*Kwoniella pini*	30173275	XP_070059334
Ssk-KSH	*Kwoniella shivajii*	87959241	XP_062794856
Ssk-KER	*Kwoniella europaea*	91100632	XP_066081739
Ssk-KSN	*Kwoniella shandongensis*	43588188	XP_031861659

Ssk-KBO	*Kwoniella botswanensis*	90016042	XP_064746845
Ssk-KEP	*Kwoniella dendrophila*	91097444	XP_066078590
Ssk-TMS	*Tremella mesenterica*	TREMEDRAFT_33972	XP_007006711
Ssk-TASA	*Trichosporon asahii* var. asahii	A1Q1_08269	XP_014181891
Ssk-CCAC	*Cutaneotrichosporon cavernicola*	CcaHIS019_0104630	XP_060453011
Ssk-WSE	*Wallemia mellicola*	WALSEDRAFT_47292	XP_006959301
Ssk-WIC	*Wallemia ichthyophaga*	J056_000430	XP_009268333
Ssk-UMA	*Mycosarcoma maydis*	UMAG_01544	XP_011387573
Ssk-PFP	*Pseudozyma flocculosa*	PFL1_05920	XP_007881646
Ssk-SGRA	*Sporisorium graminicola*	EX895_004340	XP_029738685
Ssk-MGL	*Malassezia globosa*	MGL_3138	XP_001729594
Ssk-MRT	*Malassezia restricta*	MRET_1340	XP_027483619
Ssk-MSYM	*Malassezia sympodialis*	MSY001_2850	XP_018741352
Ssk-MVES	*Malassezia vespertilionis*	80903459	XP_056064733
Ssk-PGR	*Puccinia graminis*	PGTG_03102	XP_003321565
Ssk-PTRC	*Puccinia triticina* (wheat leaf rust)	PtA15_1A832	XP_053017045
Ssk-PSTR	*Puccinia striiformis* f. sp. tritici	Pst134EA_000257	XP_047812632
Ssk-MLR	*Melampsora larici-populina*	MELLADRAFT_116464	XP_007410166
Bck1-LBC	*Laccaria bicolor*	LACBIDRAFT_378797	XP_001873764
Bck1-MORE	*Marasmius oreades* (fairy-ring mushroom)	E1B28_001025	XP_043015624
Bck1-SCE	*Saccharomyces cerevisiae* (budding yeast)	YJL095W	NP_012440
Bck1-PPL	*Postia placenta*	POSPLDRAFT_103650	EED79364
Bck1-CNE	*Cryptococcus deneoformans* JEC21	CNJ01850	XP_024513618
Bck1-CNB	*Cryptococcus deneoformans* B-3501A	CNBJ1620	XP_773167
Bck1-CNG	*Cryptococcus neoformans* var. grubii H99	CNAG_04755	XP_012052184
Bck1-CGI	*Cryptococcus gattii*	CGB_I2500W	XP_003196136
Bck1-CDEU	*Cryptococcus deuterogattii*	CNBG_9651	XP_062885998
Bck1-CDEC	*Cryptococcus decagattii*	89992039	XP_064723150
Bck1-CTEG	*Cryptococcus tetragattii*	91992391	XP_066611936
Bck1-CBAS	*Cryptococcus bacillisporus*	91983507	XP_066607388
Bck1-CDEP	*Cryptococcus depauperatus*	91088761	XP_066070029
Bck1-KMG	*Kwoniella mangroviensis*	30161612	XP_019004956
Bck1-KNE	*Kwoniella newhampshirensis*	92182650	XP_066801052
Bck1-KDJ	*Kwoniella dejecticola*	28965374	XP_018265312
Bck1-KBI	*Kwoniella bestiolae*	30207979	XP_019046974
Bck1-KPIN	*Kwoniella pini*	30175253	XP_019008327
Bck1-KSH	*Kwoniella shivajii*	87953689	XP_062789365
Bck1-KER	*Kwoniella europaea*	91104377	XP_066085442
Bck1-KSN	*Kwoniella shandongensis*	43591642	XP_065823846
Bck1-KBO	*Kwoniella botswanensis*	90018653	XP_064749430
Bck1-KEP	*Kwoniella dendrophila*	91092279	XP_066073492
Bck1-TASA	*Trichosporon asahii* var. asahii	A1Q1_07181	XP_014182892
Bck1-UMA	*Mycosarcoma maydis*	UMAG_01662	XP_011387646

Table S5. Sequence identity between LeMAPKKKs and their yeast homologs (Ssk, Bck1, and Ste11)

Percent Identity	LeMAPKKK1	LeMAPKKK2	LeMAPKKK3
Ste11-ABP	34.60%	38.22%	33.59%
Ste11-ADL	34.08%	39.47%	35.63%
Ste11-CBAS	33.59%	36.84%	33.72%
Ste11-CCAC	33.08%	36.40%	33.58%
Ste11-CCI	34.34%	37.74%	34.42%
Ste11-CDEC	33.21%	36.84%	33.72%
Ste11-CDEP	35.11%	36.88%	34.33%
Ste11-CDEU	33.97%	37.22%	34.10%
Ste11-CGI	33.59%	37.22%	33.72%
Ste11-CNB	34.35%	37.97%	34.10%
Ste11-CNE	34.35%	37.97%	34.10%
Ste11-CNG	33.59%	37.40%	34.87%
Ste11-CPUT	33.70%	39.55%	34.10%
Ste11-CTEG	33.97%	36.84%	34.10%
Ste11-DSQ	32.70%	36.01%	34.13%
Ste11-FME	34.22%	37.36%	37.40%
Ste11-GTR	32.14%	39.16%	34.10%
Ste11-HIR	33.84%	35.58%	33.08%
Ste11-KBI	35.11%	37.88%	33.83%
Ste11-KBO	35.11%	37.88%	33.33%
Ste11-KDJ	34.73%	37.12%	34.22%
Ste11-KEP	35.11%	37.88%	34.22%
Ste11-KER	35.11%	37.88%	33.69%
Ste11-KMG	35.11%	37.50%	34.57%
Ste11-KNE	33.97%	38.02%	32.83%
Ste11-KPIN	35.11%	37.50%	34.60%
Ste11-KSH	34.73%	37.12%	35.23%
Ste11-KSN	33.59%	38.02%	32.46%
Ste11-MGL	33.83%	38.26%	35.00%
Ste11-MORE	35.11%	35.36%	34.48%
Ste11-MRR	35.88%	35.36%	34.41%
Ste11-MRT	33.82%	37.69%	32.95%
Ste11-MVES	34.52%	37.45%	35.91%
Ste11-PFP	34.70%	36.00%	36.40%
Ste11-PGR	34.85%	37.12%	34.73%
Ste11-PSQ	32.95%	37.36%	32.06%
Ste11-PSTR	34.35%	37.26%	34.33%
Ste11-PTRC	33.97%	36.88%	35.25%
Ste11-RSX	33.33%	37.05%	34.98%
Ste11-SCE	37.39%	31.79%	35.02%
Ste11-SCM	33.21%	36.50%	32.95%
Ste11-SGRA	32.50%	34.27%	34.07%
Ste11-SHS	33.21%	37.17%	33.72%
Ste11-TASA	34.21%	36.96%	33.85%
Ste11-UMA	33.57%	34.04%	34.80%
Ste11-WIC	34.43%	37.54%	32.72%
Ste11-WSE	34.59%	38.91%	34.34%
Ssk22-SCE	31.34%	33.45%	31.48%
Ssk2-SCE	30.69%	32.67%	30.07%

Ssk-ABP	34.15%	34.25%	30.42%
Ssk-ABV	33.33%	33.33%	27.75%
Ssk-ADL	33.94%	37.37%	32.08%
Ssk-CBAS	32.84%	31.29%	33.33%
Ssk-CCAC	32.46%	35.59%	30.29%
Ssk-CCI	33.57%	35.31%	28.70%
Ssk-CDEC	33.21%	31.65%	33.71%
Ssk-CDEP	32.46%	32.01%	32.96%
Ssk-CDEU	32.84%	31.65%	33.71%
Ssk-CGI	33.21%	31.65%	33.71%
Ssk-CNB	32.46%	32.01%	32.58%
Ssk-CNE	32.46%	32.01%	32.58%
Ssk-CNG	32.84%	32.37%	32.96%
Ssk-CPUT	33.22%	34.72%	28.88%
Ssk-CTEG	33.21%	31.65%	33.71%
Ssk-DSQ	29.29%	33.90%	30.25%
Ssk-FME	33.33%	35.71%	31.21%
Ssk-GTR	33.33%	35.34%	31.14%
Ssk-HIR	31.25%	36.49%	31.25%
Ssk-KBI	32.22%	31.18%	30.97%
Ssk-KBO	31.60%	31.54%	31.34%
Ssk-KDJ	31.97%	31.54%	32.09%
Ssk-KEP	31.23%	31.54%	31.34%
Ssk-KER	31.60%	31.54%	31.34%
Ssk-KMG	31.23%	31.18%	30.97%
Ssk-KNE	31.97%	32.26%	32.46%
Ssk-KPIN	31.97%	31.90%	32.09%
Ssk-KSH	31.97%	32.26%	31.34%
Ssk-KSN	32.09%	32.37%	32.28%
Ssk-LBC	33.33%	34.83%	29.14%
Ssk-MGL	32.86%	36.97%	30.47%
Ssk-MLR	34.04%	35.93%	32.08%
Ssk-MORE-1	32.37%	36.36%	30.71%
Ssk-MORE-2	33.09%	35.69%	30.18%
Ssk-MRR	31.36%	34.60%	29.03%
Ssk-MRT	33.33%	35.66%	30.70%
Ssk-MSYM	31.64%	35.82%	29.12%
Ssk-MVES	29.10%	34.95%	29.93%
Ssk-PCO	31.79%	35.76%	30.07%
Ssk-PFP	31.72%	34.67%	28.72%
Ssk-PGR	32.33%	34.22%	31.27%
Ssk-PPL-1	29.43%	32.19%	27.51%
Ssk-PPL-2	30.60%	31.90%	27.91%
Ssk-PSQ	34.40%	35.71%	30.14%
Ssk-PTRC	32.16%	34.26%	31.41%
Ssk-RSX	33.10%	32.40%	31.43%
Ssk-SCM	30.99%	36.36%	27.49%
Ssk-SGRA	31.25%	33.22%	28.06%
Ssk-SHS	33.69%	34.97%	32.85%
Ssk-SLA	31.71%	35.54%	29.47%
Ssk-TASA	28.32%	34.53%	30.96%
Ssk-TMS	29.01%	32.73%	29.66%

Ssk-TVS	29.93%	34.38%	31.36%
Ssk-UMA	31.25%	32.21%	28.35%
Ssk-WIC	30.80%	35.54%	30.58%
Ssk-WSE	31.39%	36.40%	31.45%
Bck1-CBAS	34.49%	36.40%	32.36%
Bck1-CDEC	34.49%	36.40%	32.36%
Bck1-CDEP	34.67%	37.18%	31.84%
Bck1-CDEU	34.49%	36.40%	32.36%
Bck1-CGI	34.84%	36.76%	32.73%
Bck1-CNB	35.19%	36.76%	33.09%
Bck1-CNE	35.19%	36.76%	33.09%
Bck1-CNG	35.54%	37.13%	33.45%
Bck1-CTEG	34.49%	36.40%	32.36%
Bck1-KBI	32.73%	36.03%	32.51%
Bck1-KBO	32.48%	35.97%	32.16%
Bck1-KDJ	32.73%	35.29%	33.22%
Bck1-KEP	31.39%	35.61%	32.51%
Bck1-KER	32.48%	35.61%	32.16%
Bck1-KMG	32.48%	35.61%	32.16%
Bck1-KNE	34.06%	36.26%	32.54%
Bck1-KPIN	33.21%	34.53%	33.58%
Bck1-KSH	33.10%	36.03%	32.00%
Bck1-KSN	33.33%	35.90%	32.03%
Bck1-LBC	32.44%	40.15%	31.94%
Bck1-MORE	33.21%	40.22%	33.08%
Bck1-PPL	27.11%	32.76%	28.23%
Bck1-SCE	30.56%	36.78%	34.48%
Bck1-TASA	30.43%	34.72%	31.68%
Bck1-UMA	32.46%	41.06%	32.95%

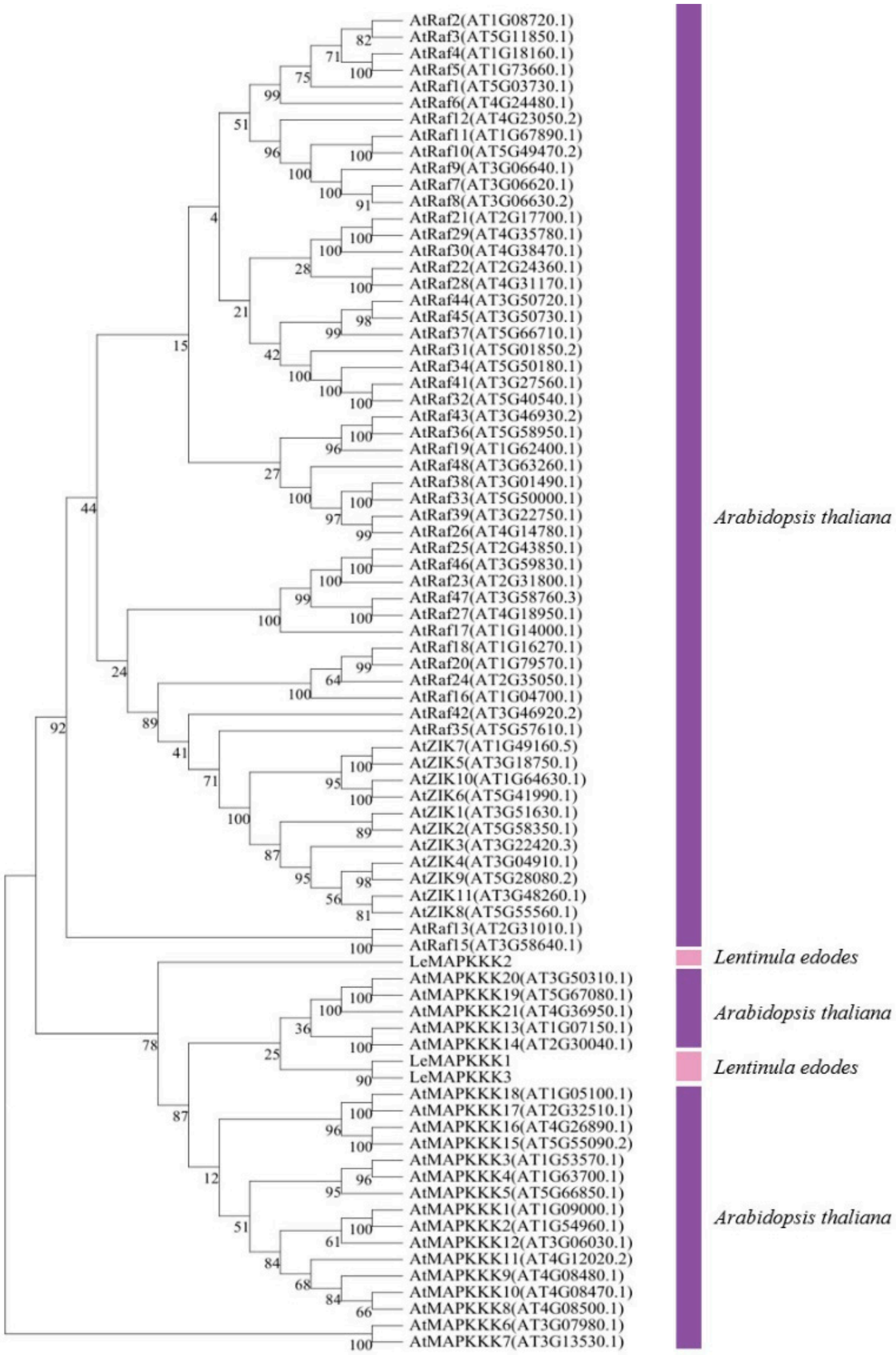

Figure S1. Phylogenetic and homology analysis of LeMAPKKKs in *L. edodes* and *Arabidopsis thaliana*.

The phylogenetic tree shows the clustering patterns of two *L. edodes* LeMAPKKKs with MAPKKK homologs in *A. thaliana*. *L. edodes* sequences (LeMAPKKKs) are highlighted in pink, and *A. thaliana* homologs are shown in purple. *A. thaliana* sequences were obtained from The Arabidopsis Information Resource (TAIR). The phylogenetic tree was constructed using MEGA 11 with the NJ method.
